# Theoretical Methods of Domain Structures in Ultrathin Ferroelectric Films: A Review

**DOI:** 10.3390/ma7096502

**Published:** 2014-09-12

**Authors:** Jianyi Liu, Weijin Chen, Biao Wang, Yue Zheng

**Affiliations:** 1 State Key Laboratory of Optoelectronic Materials and Technologies, School of Physics and Engineering, Sun Yat-sen University, Guangzhou 510275, China; E-Mails: liujyi2@mail2.sysu.edu.cn (J.Y.L.); chenwjin@mail2.sysu.edu.cn (W.J.C.); wangbiao@mail.sysu.edu.cn (B.W.); 2 Micro & Nano Physics and Mechanics Research Laboratory, School of Physics and Engineering, Sun Yat-sen University, Guangzhou 510275, China

**Keywords:** domain structure, theoretical methods, simulation, ultrathin ferroelectric film

## Abstract

This review covers methods and recent developments of the theoretical study of domain structures in ultrathin ferroelectric films. The review begins with an introduction to some basic concepts and theories (e.g., polarization and its modern theory, ferroelectric phase transition, domain formation, and finite size effects, *etc.*) that are relevant to the study of domain structures in ultrathin ferroelectric films. Basic techniques and recent progress of a variety of important approaches for domain structure simulation, including first-principles calculation, molecular dynamics, Monte Carlo simulation, effective Hamiltonian approach and phase field modeling, as well as multiscale simulation are then elaborated. For each approach, its important features and relative merits over other approaches for modeling domain structures in ultrathin ferroelectric films are discussed. Finally, we review recent theoretical studies on some important issues of domain structures in ultrathin ferroelectric films, with an emphasis on the effects of interfacial electrostatics, boundary conditions and external loads.

## 1. Introduction

Ferroelectrics are a certain family of materials possessing spontaneous polarization in a definite range of temperature, which can be reversed, in particular, by the application of an external electric field [1,2]. When a paraelectric ferroelectric is cooled down through the phase transition temperature, *i.e.*, the Curie temperature, a domain structure consisting of different domain variants would be formed, which is one of the most distinctive features of ferroelectric materials. In the literature, considerable attention has been devoted to issues of ferroelectric domain structures, for the very practical reason that most of the important applications of ferroelectric materials (e.g., memories, sensors and actuators) are determined to a great extent by the stability and evolution of the domain structures. During the past decades, fundamental physics and device applications of domain structures in ferroelectric crystals have been extensively studied and well understood.

Driven by the trend of device miniaturization, integration and multi-functionalization, ferroelectric thin films have been the objects of intensive academic interests, for their original properties which are of scientific significance to understand and are promising in developing advanced functional devices. Examples include non-volatile memories [3] in microelectronics, mechanical sensors and actuators [4] in micromechanics, pyroelectric detectors [5], and tunable microwave and electro-optical devices [6], *etc.* Recently, the main focus of ferroelectric thin films has been turned to ultrathin ferroelectric films (UFFs) with the film thickness ranging from a few to dozens of nanometers. Being nanoscale in one dimension, the finite size effect is considerable to diversify the domain patterns and properties of the UFFs from those of their bulk counterparts. For example, the dielectric constants being very high in bulk material (like in SrTiO_3_ and Pb(Mg_1/3_Nb_2/3_)O_3_ (PMN)) are strongly reduced in the films, while the values of the coercive fields are elevated (e.g., in Pb(Zr,Ti)O_3_ (PZT) films) [7]. For UFFs systems, novel fundamental issues arise, e.g., how thin an UFF can be made before the ferroelectricity vanishes due to the intrinsic size effects, and how behaviors of domain structure and related properties such as transport property depend on thickness. Practically, the domains behaviors of an UFF can be attributed to the combined effect of many specific factors; surface/interface chemistry, in-plane strain, defects (e.g., point defects and dislocations), strain gradient, electrode screening, and ambient chemical environment are examples. Understanding the acting mechanisms of different factors and finding out the regularities that control the stability and evolution of domain structures are essential for academic research of UFFs and promising technological applications.

Historically speaking, Landau and Lifshitz [8], and Kittel [9] first theoretically considered the domain formation in ferromagnetic thin films. They successfully predicted the existence of the closure domains. Kittel discussed the dependence of the domain periodicity on film thickness, *i.e.*, the Kittel law in magnetic systems. This was then obtained for ferroelectric domains by Mitsui and Furuichi [10]. Based on thermodynamic analysis, various analytical models were then proposed, e.g., by Roytburd [11,12,13,14,15,16], Pertsev and Tagantsev [17,18,19,20,21,22,23] *et al.*, in the investigation of the domain structures in UFFs and heterostructures. Despite the limitation in predicting the domain structure in details and the fact that it must be applied with discretion, such as a careful choice of order parameters [7], thermodynamic approach has been widely applied in capturing the behaviors of domain structure in UFFs. Recently, with the repaid development of computer technology, computer-based simulation and calculation methods have played central roles in developing detailed understanding of the domain structures in UFFs. A variety of approaches scaling from an atomic level to mesoscale and macroscale, e.g., first-principles calculation, molecular dynamics (MD) simulation, Monte Carlo (MC) simulation, effective Hamiltonian method and phase field method, have been adopted in the investigations of ferroelectric domain structures during the past two decades. Presently, simulations on domain structures in UFFs are mainly performed on simple ferroelectrics (e.g., UFFs of perovskite type like BaTiO_3_, PbTiO_3_, and PZT, *etc.*) under various boundary conditions and their response to simple external electrical and mechanical loads [24,25,26,27], yet with a rapid extending to the domain structures in multiferroic thin films (e.g., BiFeO_3_, TbMnO_3_ and DyMnO_3_), dynamics of domain structures, functionality of domain walls such as photovoltaic effect and conductivity, *etc.*, which are all intriguing issues nowadays.

In this review, we give an account of the recent progresses achieved in understanding the domain structures in UFFs based on theoretical methods, with the main focus being on numerical approaches. The review generally consists of three sections. As a starting point, in Section 2, fundamentals of UFFs, especially those basic concepts and theories intimately related to the discussion of domain structure are reviewed, aiming at providing readers an overall knowledge of domain structures in UFFs. In Section 3, we elaborate the basic aspects and developments of various approaches for domain structure simulation, including first-principles calculations, MD simulation, MC simulation, effective Hamiltonian approach, and phase field method, with a particular attention to their technique features when tackling UFFs. The recent developments of the multiscale simulation scheme are presented, and the relative merits of each approach are also discussed. In Section 4, we review recent achievements in some important issues of domain structures in ultrathin ferroelectric films based on the theoretical studies, with an emphasis on the effects of interfacial electrostatics, boundary conditions and external loads. Finally, we conclude this review with a brief summary and outlook. 

## 2. Fundamentals of Ultrathin Ferroelectric Films (UFFs)

The fundamental theories of ferroelectrics have been developed over a long period and documented in numerous text books (see, for example, Ref. [1]). Yet most attention has been paid to infinite bulk ferroelectrics or relatively thick films (>100 nm), with a relative lacking of considerations on ultrathin films. The most essential feature of UFFs is that the inhomogeneity of properties near the surfaces or interfaces can bring significant effects to the material, namely finite size effects, which are important to understand for the continuous scaling down of ferroelectrics. In this Section, we would make a brief review on some basic concepts and theories relevant to the discussion of domain structure in UFFs, with special attention to the recent developments of these concepts and theories. As domain evolution in UFFs is ultimately attributed to the change of polarization distribution, we begin this Section with the concept of polarization and its modern theory, followed by the introduction of different models of ferroelectric phase transition, with the purpose of providing readers a background on the origin of domain structure. Then we talk about domain formation, with a review on some common types of domain patterns and regularity of domain formation. Finally, finite size effects, which play important roles in the behaviors of domain structures in UFFs, are discussed. 

### 2.1. Polarization and Its Modern Theory

A ferroelectric domain structure generally consists of different domain variants with a specific polarization vector, making it natural to use polarization field to characterize a ferroelectric domain structure. An accurate evaluation of polarization is thus important for the study of domain structures. Nevertheless, for a long time the definition of polarization was an unsolved problem. As what is done in classical electrodynamics, people used to define the macroscopic polarization via charge distribution, *i.e.*,

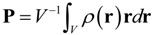
(1)
where *V* is the volume of the sample; and ρ(**r**) is the charge density due to ions and electrons. In Equation (1), the change density ρ(**r**) must obey the condition of zero total charge; otherwise a net charge must be subtracted. This definition, nevertheless, is only applicable for finite systems or perfect ionic crystals described by the Clausius-Mossotti model. For infinite polarized systems, as usually encountered in first-principles calculations and other simulation approaches, such formulation is ill-defined for its dependence on choice of unit-cell. Moreover, it is difficult to implement as the absolute value of polarization cannot be measured in experiment. 

What actually measured in experiment is the polarization change Δ**P** of the sample. Noting this and the fact that many polarization-related quantities of interests (e.g., dielectric, pyroelectric and piezoelectric tensors) are polarization derivatives, Resta [28] took a pioneering step to identify Δ**P** as a fundamental quantity and derived a well-defined form of Δ**P** under the framework of density-functional perturbation theory (DFPT). Subsequently, King-Smith and Vanderbilt [29] linked it with a certain geometric phase known as Berry phase [30,31], making it computationally efficient [32,33]. The development of this modern theory of polarization has been reviewed by Resta [34,35] and discussed in detail by Rabe *et al.* [36]. In this theory, the polarization change is written as:

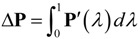
(2)
where *λ* is a dimensionless parameter varying continuously from zero (the initial state) to one (the finial state); and **P***'*(*λ*) is the change rate of **P** with respect to *λ*. Accordingly, spontaneous polarization of ferroelectrics can be defined by choosing *λ* as displacement of the lattice structure varying from the centrosymmetric structure to polarized structure under a vanishing external field.

Based on the Born-Oppenheimer approximation, Δ**P** can be decomposed into contributions from the atom nuclei and the electrons. The contribution from the atom nuclei can be calculated as:

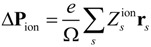
(3)
with *e* being the electron charge; Ω the volume of the unit cell; and 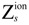 the charge of the nucleus located at the position **r***_s_*. Based on the first order adiabatic perturbation theory, the electron contribution can be deduced as:

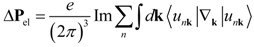
(4)
where *u_n_***_k_** is periodic function related to the Bloch wave function as *ψ_nk_* = *e^i^*^**k** · **r**^*u_n_*_**k**_; ***k*** the wave vector; and *n* labels the energy level. The integral in Equation (4) is independent of the path and is closely related to the Berry’s phase (for a review of physical manifestations of Berry’s phase, see [37]). Owing to this reason, the modern theory of polarization is also known as Berry phase method. The modern theory of polarization largely facilitates the study of ferroelectrics based on first-principles calculations. The polarization value of bulk ferroelectrics, the energy dependence on polarization, polarization profiles at the domain walls or near surfaces and interfaces, *etc.*, can be all readily and accurately obtained from first-principles calculations. This is also important for those first-principles-based approaches, e.g., *ab initio* MD simulation, effective Hamiltonian approach and first-principles-based phase-field model, due to the need of extracting polarization-related parameters from first-principles calculations.

### 2.2. Ferroelectric Phase Transition

Typically, ferroelectric materials exhibit ferroelectricity and domain structure only below a certain temperature point, called Curie temperature. A ferroelectric phase transition is generally a structural phase transition, as the spontaneous polarization occurs with a structural distortion from a high symmetry paraelectric phase to a low symmetry ferroelectric phase. Ferroelectric phase transition can be divided into displacive type (e.g., ferroelectric perovskites with oxygen octahedrons like BaTiO_3_ and PbTiO_3_) and order-disorder type (e.g., composites with hydrogen bonds like KH_2_PO_4_ (KDP)), according to whether the symmetry change is caused by collective displacement of sublattices or order-disorder redistribution of dipoles over equiprobable directions. Nevertheless, many materials (e.g., BaTiO_3_ and PbTiO_3_) are likely to have both displacive and order-disorder features. Moreover, a ferroelectric phase transition can be either first-order type or second-order type depending on whether the spontaneous polarization continuously changes across the phase transition point. 

In literature, various theoretical models have been established to capture the ferroelectric phase transition behaviors. Among them, the most famous ones are the phenomenological Landau theory [38,39,40,41], soft mode theory [42,43,44,45] and Ising model [46,47]. The former one is usually called as a macroscopic model, and the latter two are called as microscopic models, according to whether the theory concerns with the microscopic origin of ferroelectric phase transition. In general, Landau theory is applicable to most of the ferroelectric systems as it is based on a universal symmetry argument. Meanwhile, soft mode model mainly deals with displacive type ferroelectric phase transition, and Ising model mostly applies for order-disorder type ferroelectric phase transition. Note that, based on these phase transition models, simulation approaches of ferroelectric domain structure can be further developed by extending the homogenous case to inhomogeneous case. Typical examples include phase field method based on Landau theory, effective Hamiltonian approach based on soft-mode theory and Monte Carlo simulation based on the Ising model. 

Before a review on the three ferroelectric phase transition models, we would like to point out the relation between them. In fact, Landau theory is essentially a mean-field theory, meanwhile the latter two usually work within a mean-field approximation [47,48]. As a consequence, they generally give an equivalent description of ferroelectric phase transition. Specifically, the order parameter characterizing the phase transition in Landau potential is exactly related to the thermal average of the soft mode coordinate (thermal average of the *z* component of the pseudo-spin). It should be noted that mean-field theories give a qualitatively correct view of the ferroelectric transitions but break down when the fluctuations are significant. Fortunately, in contrast to ferromagnetic phase transition, the long-range interaction that tends to suppress fluctuations plays a dominant role in ferroelectric ordering. Therefore, the critical region is actually rather small, and mean-field models are believed to be reliable for ferroelectric transitions. Levanyuk and Ginzburg [49,50] discussed the fluctuations of the order parameter and developed a criterion for the validity of Landau theory. For soft mode theory and Ising model, the fluctuations are usually taken into account within the framework of effective field theory with a probability distribution technique taking into account the spontaneous polarization or self-spin correlation function [47,51].

#### 2.2.1. Phenomenological Theory of Ferroelectric Phase Transition: Landau Theory

Landau [38,39] noted that structural phase transitions are related to symmetry broken in the low temperature phases characterized by the spontaneous appearance of the order parameter, and suggested that the free energy of the system in the vicinity of phase transitions can be expanded as a power series of order parameter. This was then first applied to the study of BaTiO_3_ crystals by Devonshire [40,41] and extended to the case of antiferroelectric crystals by Kittel [52] and Devonshire [53]. For the simple case of a homogeneous and uniaxial ferroelectric system, the Landau free energy density in absence of external electric and mechanical fields can be expressed up to a sixth-order polynomial as:

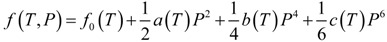
(5)
Here the polarization *P* is taken as the order parameter; *a*(*T*), *b*(*T*) and *c*(*T*) are expansion coefficients that are generally temperature dependent. The even terms are eliminated due to the symmetry compatibility. The equilibrium value of the spontaneous polarization can be determined by:

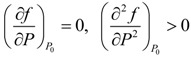
(6)


The coefficient *a*(*T*) is always assumed to be *a*(*T*) = *a*_0_(*T* − *T*_0_) with *a*_0_ > 0 being constant and *T*_0_ the ferroelectric transition temperature, so that a solution *P* = 0 (corresponding to the paraelectric phase) is stable at high temperature and a solution *P* ≠ 0 (corresponding to the ferroelectric phase) is stable at low temperature. Moreover, *a*_0_, *T*_0_, *b* and *c* can be determined by fitting to the experimental data or first-principles calculations. Note that the order of ferroelectric phase transitions can be characterized by the sign of the coefficient *b*. If *b* > 0, it characterizes a second-order ferroelectric phase transition, whereas the transition is first-order if *b* < 0. To describe the dynamic process, an irreversible thermodynamic equation is usually used as ∂*P*/∂*t* = −*M* ∂*f*/∂*P*, where *M* is a kinetic coefficient and *t* is time. This equation is based on the intuitional fact that the system is driven to equilibrium by a “thermodynamic force” ∂*f*/∂*P*. 

A more detailed discussion on the Landau theory of ferroelectric phase transition can be found in the book of Fatuzzo and Merz [54], the review paper by Müser and Petersson [55], and the book of Blinc and Žekš [47]. Note that the Landau theory has its limitations. Firstly, as discussed above, being a mean-field theory, Landau theory ignores order parameter fluctuations. This limitation is often discussed in literature, and a good discussion can be found in the book of Strukov and Levanyuk [56]. Secondly, the use of a macroscopic polarization as a variable for the Landau expansion, *i.e.*, order parameter, is not fully justified from the point of view of the microscopic theory. One may use quantities such as electric displacement [1], total polarization [57,58], spontaneous polarization [59,60], ionic polarization caused by soft mode displacement [61], and soft-mode displacement within the weak-ferroelectric approach [62], *etc.*, as the order parameter. In many cases, particularly in bulk systems, the specific choice of the order parameter makes no significant difference. However, in some cases, such as UFFs under open-circuit condition, an improper choice of the order parameter can lead to unreliable results. Thirdly, being a continuum theory, a limitation resides in the rationality of Landau theory when dealing with domains and domain walls with fairly small size. Finally, as a phenomenological theory, its accuracy largely depends on the complexity of the potential and its parameters, the accuracy of which are sensitive to the experiment data or first-principles calculations. Moreover, since the parameters are determined within a specific condition, the rationality of an extrapolation to other conditions is often questioned. Despite its limitations, for its universality and simple formulism, Landau theory has been widely applied to describe the phase transition behaviors of a large amount of ferroelectric materials. During the last two decades, phase field method based on the Landau theory has been developed rapidly to simulate the evolution of ferroelectric domain structure. A review on phase field method will be provided in Section 3.5. 

#### 2.2.2. Microscopic Theory of Ferroelectric Phase Transition

In literature, the microscopic theories for ferroelectric transitions of displacive type and order-disorder type have been discussed, mainly basing on the soft mode theory and the Ising model, respectively. In the following we briefly introduce the two models. It should be noticed that ferroelectric phase transitions in many crystals actually have been observed to possess characteristics of both displacive type and order-disorder type. For instance, hints for order-disorder type were found in the ferroelectric transitions of BaTiO_3_ and PbTiO_3_, which have been considered as typical displacive type [63,64,65,66,67]. This promoted the development of the unification of theories [68,69,70]. On the other hand, the contribution of electrons was also taken into account in later investigations [71]. In partcular, first-principles calculations shed enormous light on understanding nature of ferroelectric transitions [72,73,74,75].

While the thermodynamics of ferroelectric phase transition based on Landau theory has been well-established in early 1950s, the development of microscopic theory of ferroelectric phase transition did not get any significant breakthrough until 1960s. Cochran [42,43,44] and Anderson [45] independently proposed the concept of soft mode to give an atomistic level picture of dynamic mechanism of ferroelectric phase transitions. They suggested that the phase transitions in certain crystals are actually the result of instability of the crystal for a certain normal mode of vibration, and can be treated as a problem in lattice dynamics. Great advances were achieved soon after this, and the existence of soft mode was experimentally confirmed by Raman scattering [76], infrared [77], neutron scattering [78], *etc.* One can refer to the publications of Blinc [79] and Venkataraman [80] for a brief history and discussion about the soft mode concept.

In this theory, the frequency of the relevant soft phonon decreases on approaching the critical temperature *T*_0_ with:

*ω*^2^ (**q**) = *K* (*T* − *T*_0_) (7)

and the restoring force for the mode tends to zero until the phonon has condensed out at the stability limit. This can be attributed to the competition between the short-range interactions favoring paraelectric phase and the long-range Coulomb force preferring ferroelectric ordering. They are temperature-dependent and cancel with each other at *T*_0_, leading to a phase transition. Specifically, the static displacement on going in the structural phase transitions corresponds to the frozen-in mode displacement of the unstable phonon, and the physical quantity characterizing the phase transition is the static component of the eigenvector of the soft mode. The ferroelectric ordering involves the softening of certain transverse polar modes at the center of the Brillouin zone.

Soft mode theory provides a natural conceptual framework for the numerical approaches of ferroelectricity such as first-principles calculations and effective Hamiltonian method. It is worth noting that the soft mode theory was meant for ferroelectric transitions of displacive type like BaTiO_3_ at the very beginning. Then later, the basic idea was found applicable to the order-disorder systems like KDP by taking the unstable pseudo-spin waves rather than phonons as the collective excitation. 

For phase transitions of order-disorder type, typically hydrogen bonded systems where the protons can move between the two equilibrium sites in the H-bond potential, Gennes [46] and Blinc and Zeks [47] treated a ferroelectric as a system of pseudo-spins with interactions. In the limit of a steep double well potential, the model Hamiltonian of the pseudo-spin formalism can be written as:

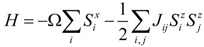
(8)
where *S_i_^x^* and *S_i_^z^* are the *x* and *z* components of the pseudo-spin lactated at site *i*, respectively; and Ω is the proton tunneling frequency between the two equilibrium positions on the H bonds; *J_ij_* is the coupling coefficient describes the interaction between pseudo-spin lactated at site *i* and *j*. Note that the model Hamiltonian in Equation (8) is exactly the same as the Ising-type Hamiltonian in a transverse field if considering Ω as a transverse field, thus known as the transverse Ising model (TIM). Furthermore, it is noteworthy that Equation (8) is meant for second-order phase transitions. An additional term of four-spin interactions should be included when considering the first-order phase transitions [81,82].

TIM was then successfully applied to many other systems [83,84] and extended to study properties of ferroelectric thin films by Wang *et al.* [48,82,85,86,87] and Sy [88]. They modified the tunneling frequency and pseudo-spin interaction near the surface to include the size effects. In partcular, Wang *et al.* [86] considered conditions with long-range pseudo-spin interactions and assumed it to be the form as *J_ij_* = *J*_1_/*r_ij_^δ^*, where *r_ij_^δ^* is the distance between site *i* and *j*, and *δ* is the decaying exponent. However, the materials of most of the interesting functional ferroelectric films are typically displacive. For the ferroelectrics with a displacive phase transition such as BaTiO_3_, Oubelkacem *et al.* [89] suggested that Ω possibly represents a multiplicity of the Ti positions inside the oxygen cage. To the best of our knowledge, so far there is no calculation of ferroelectric domain structures based on TIM. Meanwhile, there are investigations of domain switching based on Potts model, which is a direct extension of the Ising model. A review of this will be given in Section 3.3.

### 2.3. Domain Formation

In this part, we proceed to the vital concept of ferroelectric domain structures. Generally, regions with uniform electric polarization are known as ferroelectric domains, and a system of domains with different orientations forms the so-called domain structure. When a ferroelectric is cooled down through the Curie temperature, energetically degenerated domain variants form from the parent phase with approximately equal volume fractions. The domain variants are separated by the boundaries called ferroelectric domain walls, which are usually extremely thin regions with the thickness ranging from one to several tens of lattice units. Essentially, ferroelectrics form domain structures to reduce the electrostatic and elastic energy stored in the ferroelectric system. Domain formation is thus largely affected by many factors that are closely connected with the electrical and elastic influences (e.g., phase transition, crystalline state, defects, external fields, and boundary conditions, *etc.*). 

From a general point of view, the equilibrium domain structure in a ferroelectric is determined by minimization of the total free energy of the system, which is typically a sum of electrostatic energy, elastic energy, and domain wall energy, *etc.* [90,91]*.* The reduction of electrostatic and elastic energy is favored if the domain is aligned along the electrostatic field and its lattice strain matches the applied stress, while the existence of domain wall would contribute a positive energy. For example, the experimentally observed ferroelastic domain patterns (*i.e.*, *a*/*c*- and *a*1/*a*2- variants as well as their superposition) in (001) ultrathin film of tetragonal ferroelectric perovskites are typically driven by the elastic effect [92]. It is noteworthy that there are domain patterns that deviate from the electrostatic or elastic compatible feature. The labyrinth and zigzag domain structures [93,94], where individual domain wall may not fulfill the electrical and elastic compatibility, are examples. This happens, especially when domains are dense enough, the additional bound charges or elastic energy may be compensated by other factors and the electrical and elastic compatibility are met in average. Moreover, topological defects, e.g., vortex and anti-vortex states [95], bubble phase [96], and spiral domain structure [97], are also discovered in UFFs. All these domain structures give rise to abundant existing and potential applications of UFFs. 

In the following, we discuss the electrostatic and elastic influences on the domain formation, with a special attention to the UFFs. 

#### 2.3.1. The Formation of 180° Domain Pattern: Role of Depolarization Field

One well known reason for forming domain structure is to decrease depolarizing energy. Note that the depolarization field can occur in ferroelectric phase due to the bound charges near surfaces/interfaces or defects of the ferroelectric, contributing to the total energy by [1]:

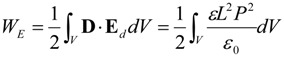
(9)
where *L* is the depolarization factor depending on the shape and polarization of the sample. In order to reduce the depolarization energy, domain structures tend to form, especially in ferroelectrics with few free charge carriers since the bound charges cannot be effectively compensated. Therefore, arbitrarily oriented domain wall which give rise to bound charges is not energetically favorable. For a pair of ferroelectric domains denoted by A and B, with corresponding spontaneous polarization being **P**_A_ and **P**_B_, the domain wall orientation must satisfy:

(**P**_A_ − **P**_B_) · **n** = 0 (10)

to maintain an electrical neutral state which is energetically favorable. Here **n** is the normal vector of the domain wall. In the absence of free carriers and elastic domains, the electrical compatibility plays a crucial role in determining the domain wall orientation. One of the most common patterns consistent with the electrical compatibility is the so-called 180° domain wall in tetragonal ferroelectric crystals.

#### 2.3.2. Elastic Compatible Domain Pattern

Mechanical compatibility can also impose severe restrictions on the formation of domain patterns. Governing by minimizing the elastic energy, the orientation of domain wall are required to make sure the transformation of any geometrical figure, lying in the plane of the wall, due to deformations of two neighboring domains caused by the spontaneous strain should be identical up to a rigid body motion. For example, let the strain of the domains be **ε**(A) and **ε**(B), respectively, then mathematically, any vector d**s** within the permissible wall fulfills the condition [98,99]

[ *ε_ij_* (*A*) − *ε_ij_* (*B*)]d*s_i_*d*s_j_* = 0 (11)

The mechanical compatibility of domains has been discussed by Janovec [100] and has been elaborated in the book by Tagantsev, Cross and Fousek [7]. Domain walls oriented so that Equation (11) is met are called stress-free domain walls, e.g., the so-called 90° domain wall in tetragonal ferroelectric crystals.

There are various domain patterns which satisfy the electrical and mechanical compatibility in Equations (10) and (11) existing in various ferroelectric materials with different symmetry. General speaking, 90° and 180° domain patterns are often adopted in tetragonal phase ferroelectrics, whereas in orthogonal phase, 60° and 120° domain patterns are also available besides the 90° and 180° domain patterns. Moreover, rhombohedral phase ferroelectrics such as BiFeO_3_ adopt 71° and 109° domain patterns besides 180° domain patterns [101,102]. Moreover, it is noteworthy that in most ferroelectric crystals, e.g., BaTiO_3_ or PZT the elastic energy usually plays a dominant role [90]. This, however, is not the case in UFFs, where the depolarization field in UFFs is significant strong and even prevails. The domain structure in an UFF is fairly sensitive to the electrical and mechanical boundary conditions. 

#### 2.3.3. Domain Structures in Ultrathin Ferroelectric Films

Domain structures in UFFs differ from those of bulk ferroelectrics in many aspects. Basically, the nature of their small thickness gives rise to quite strong finite size effects which are originally ignorable in their bulk counterparts, and leads to the unique important role of boundary conditions in determining domain structures in an UFF. UFFs suffer rich electrical and mechanical boundaries, and even small changes in boundary conditions can produce great response in ground state of domain patterns. 

For an UFF deposited on a thick substrate with different lattice parameters or thermal expansion behaviors, the UFF always tries to become coherently strained to match the lattice parameters of the underlying substrate, *i.e.*, experience a misfit strain, reducing the interface energy at the expense of an additional elastic energy. This determines the typical mechanical boundary condition in an epitaxial UFF with the fixed in-plane components of the deformation while the film is free in the out-of-plane direction. When the price of elastic energy paid for reducing interface energy is too expensive, misfit dislocations occur to decrease the strong elastic energy by relaxing the epitaxial thin film [11]. As a result, the UFF is in inhomogeneous strain state. Besides misfit dislocations, other factors such as point defects should also lead to local inhomogeneity and be important in determining the domain structure of UFFs. 

As for the effects of electrical boundary condition (see a detailed discussion in Section 2.4.2), a depolarizing field arising from the incomplete screening of bound charges can suppress ferroelectricity and govern domain formation in an UFF. The bound charges can be compensated by screening of free charge carriers from electrodes or ionic absorption form the ambient, or largely decreased by rotation of polarization. UFF in the absence of any screening, *i.e.*, suffering open-circuit (OC) boundary condition, tends to rotate polarization at the surface forming domains with vanishing total out-of-plane polarization to decrease the bond charges. Moreover, microstructure of an UFF at the surface or interface, which is usually neglected in thermodynamics models, contributes to the electrical and mechanical boundaries and has obvious effect on the domain structures in UFFs. For instance, the BaO- and TiO_2_- terminated BaTiO_3_ film make different contributions due to the differences in corresponding chemical bonds and surface tension. We will come back to the effects of boundary conditions in Section 4.

Theoretically describing effect of mechanical boundary conditions of domain formation in UFFs by thermodynamic models was the object of numerous articles [11,103,104,105,106,107]. In partcular, Pertsev *et al.* [17] developed a phenomenological theory using a transformed thermodynamic potential for epitaxial ferroelectric thin films. Based on this thermodynamic scheme, calculations were performed for BaTiO_3_ [18], PbTiO_3_ [17,19], PZT [20], and SrTiO_3_ [21], resulting in “misfit strain-temperature” phase diagrams for single-domain states of these films and revealing the important role of misfit strain in inducing new domain states and enhancing ferroelectricity, which were experimentally confirmed [108,109,110]. This thermodynamic model was then extended and adopted in the investigation of domain structures in UFFs with anisotropic in-plane misfit strains or homogeneous external mechanical loads [23,111,112,113]. Nevertheless, the absence of electrostatics-related terms, along with the assumption of single-domain and homogenous strain state, puts limit of these models on application to real situations. In literature, to account for the formation of polydomain, Pertsev and Koukhar [22] further derived a new potential enabling the determination of actual thermodynamic states inside dissimilar domains formed in epitaxial ferroelectric thin films. Roytburd [12,13,14] considered the thermodynamic principles of the formation of polydomain heterostructures due to effect of macrostresses and microstresses, and based on which, the stress dependent diagrams of stability of different polytwin microstructures were obtained. The effect of misfit dislocations on domain formation in UFFs has been taken in account in several works by Roytburd [11,13], Alpay [14,114], and Speck *et al.* [104,105]. The Kittel law, *i.e.*, the proportionality between the square of the domain width and the thickness of the film initially proposed by Kittel [9] for ferromagnetic system, was obtained for ferroelastic domains. Moreover, Balzar *et al.* [115,116] addressed the influence of inhomogeneous strain associated with crystalline defects including not only misfit dislocation but also threading dislocation and point defects. Discussion on the electrostatic effects on ferroelectric domain was pioneered by Mitsui and Furuichi [10], who reported ferroelectric domains following the Kittel law. Kopal *et al.* [117] and Bratkovsky and Levanyuk [118] investigated further the issue of domain formation in a ferroelectric capacitor (FC) containing a ferroelectric and electrode-adjacent passive layers. The equilibrium domain pattern in ferroelectric thin films on insulating substrates was discussed by Streiffer *et al.* [119]. Corresponding to various boundary conditions, thermodynamics models considering elastic and electrostatic effects and other effects such as surface or interface to be discussed in detail in Section 2.4 are adopted in lots of publications [120,121,122,123,124,125,126,127,128]. For example, based on a thermodynamic potential contributed from Landau bulk free energy, elastic energy, electrostatic energy, domain wall energy and surface energy, Chen *et al.* [127,128] calculated the domain formation in symmetric and asymmetric FCs, showing significant controllability of electrode on the domain structures.

### 2.4. Finite Size Effects

It is well known that the domain structure of ferroelectric thin films behaves quite differently from those of bulk materials, manifested with finite size effects. This is owing to the appearance of surfaces or interfaces, which result in inhomogeneity of properties in the vicinity of these regions. The finite size effects are enhanced as the film thickness decreases and become very significant in UFFs. One of the sources of finite size effects in UFFs is the depolarization field caused by the uncompensated polarization charges at the surface or interface. This is a long-range effect and can be obviously influenced by free-charges from electrodes, defects, and ambient environment, *etc.* The size effects can also be attributed to the short-range polarization variations near the surface which is expected to occur over a distance comparable to the correlation length of polarization fluctuations, known as the intrinsic surface effect.

#### 2.4.1. Polarization Relaxation near Surfaces/Interfaces

**Figure 1:**
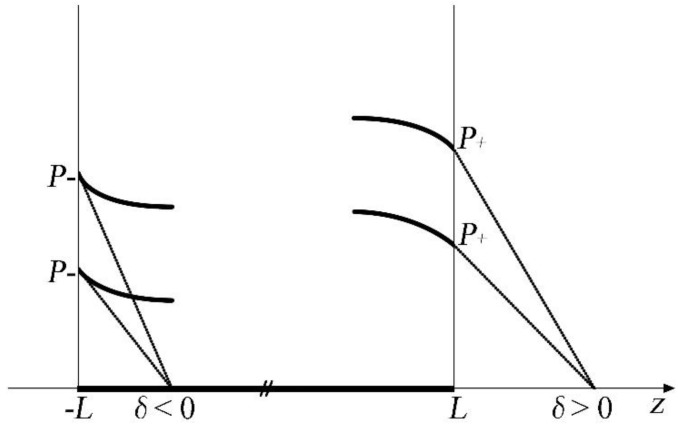
Surface polarization *P*_+_ and *P*_−_ corresponding to the extrapolation lengths *δ* > 0 and *δ* < 0 respectively. (Reproduced with permission from [133]; Copyright 2005, Springer)

Order parameter variation near surfaces/interfaces is actually a universal phenomenon, which has been originally considered in various systems including superconductors [129], superfluids [130], and magnetic systems [131]. In particular, the intrinsic surface effect on polarization in ferroelectric thin films was first investigated by Kretschmer and Binder [132] in 1979. They developed a phenomenological method to describe the polarization variations in the vicinity of ferroelectric film surface. In their approach, a surface energy governed by the surface/interface polarization and the so-called extrapolation length *δ* is added into the Ginzburg-Landau-Devonshire free energy. The surface term in Kretschmer and Binder’s framework is written as a Taylor expansion in terms polarization which includes only the second-order term. Minimizing the total free energy with respect to the polarization components naturally yields the boundary condition:

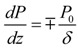
(12)
the extrapolation length can be either positive or negative. For positive extrapolation length, the polarization is reduced at the surfaces/interfaces and the Curie temperature is lower than the bulk value, whereas when the extrapolation length is negative, polarization is enhanced at the surface and it can persist well above the bulk Curie temperature as shown in Figure 1 adopted from the book by Okuyama and Ishibashi [133]. The concept of extrapolation length has been further discussed and developed by Tilley and Zeks [134].

The introduction of extrapolation length has largely extended the applicability of phenomenological approach to finite size systems. However, it should be noticed that the vulnerability of this approach also resides, considering that fact that determining the value or even the influencing factors of the extrapolation length remains difficult. Theoretical works based on the Ginzburg-Landau-Devonshire (GLD) theory usually predicted larger critical thickness of ferroelectricity in UFFs [135] compared to what has been observed experimentally. To determine the extrapolation length, Zhong and co-workers [48,82,85,86,87] have made some tentative discussion of the surface effects in ferroelectric thin films using the Ising model. Morozovska and co-workers [136,137] considered the surface energy via surface tension, making it possible to express the extrapolation length via the surface tension coefficient. They attributed the deformation tensor to surface polarization and mismatch effect (due to the differences in substrate and film lattice constants and thermal expansion coefficients) and rewrote the boundary condition in Equation (12) as:

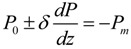
(13)
where *P_m_* is the additional polarization due to the mismatch effect at interfaces, which vanishes and Equation (13) turns back to Equation (12) for free surfaces. 

Recently, first-principles calculations shed enormous light on nature of the surface effects and yield a great deal of insights into them. The short-range intrinsic surface effect is considered to be originated from the dangling atomic bonds at the free surface or bonding of the interfacial atoms of the ferroelectric thin film to adjacent atoms in the substrate or electrode, thus of course strongly depends on the surface chemistry such as terminations. Duan *et al.* [138] performed a first-principles study of interface effect of ultrathin KNbO_3_ ferroelectric films kept between two metal electrodes and determined the value of the extrapolation length by fitting the results to the Ginzburg-Landau-Devonshire free energy. They showed that the extrapolation length is inversely proportional to the strength of bonding at the interface. Tagantsev *et al.* [122,123] developed a multiscale scheme based on the thermodynamics and first-principles calculations. Furthermore, first-principles based atomistic-level approaches are expected to play vital roles in investigating surface effects in ferroelectric thin films and determining the extrapolation length combining with the phenomenological method. However, few related works have been published nowadays [139,140,141]. This may mainly owe to the weakness in the interatomic potential model (e.g., the charge transfer which is critical in modeling surface/interface systems is not incorporated in the well-known shell model) in molecular dynamics simulations or the model Hamiltonian (e.g., most publications are based on oversimplified models) in the effective Hamiltonian method.

#### 2.4.2. Interfacial Electrostatics

The depolarization field produced by the bond polarization charges accumulated on surfaces or interfaces is another important factor in determining the finite size effects. It is considered to play predominated role in controlling ferroelectric patterns. The uniform polarization state becomes unstable and may evolve into a polydomain state to avoid the otherwise strong depolarization field. Therefore, the depolarization field can be largely reduced by formation of domain structures. Moreover, the depolarization field can be significantly influenced by the electrostatic boundary conditions. 

**Figure 2:**
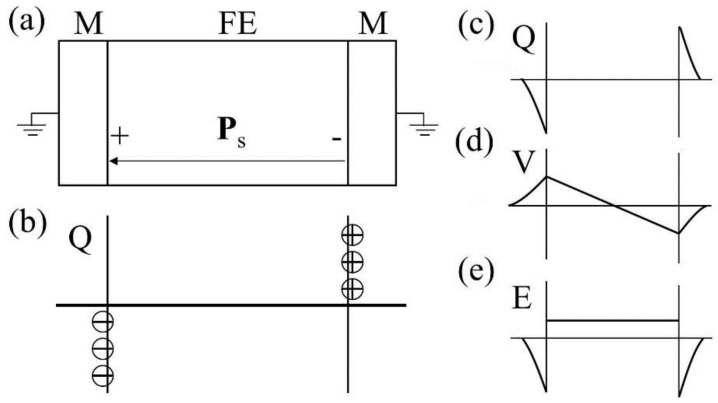
(**a**) Schematic representation of a short-circuit (SC) ferroelectric capacitor (FC) structure with the ferroelectric film homogeneously polarized with spontaneous polarization P_s_; (**b**) Schematic representation of the associated charge distribution in the presence of perfect electrodes; (**c**) Charge distribution; (**d**) voltage and (**e**) field profiles in the presence of realistic electrodes. Here, the film is taken to be a perfect insulator. (Reproduced with permission from [143]; Copyright 2003, IOP Publishing)

In an idealized ferroelectric capacitor (FC) where the electrodes are perfect conductors while the UFF is taken to be a perfect insulator as depicted in Figure 2a,b, the screening charges at the electrode/ferroelectric interface completely compensate the bond polarization charges, resulting in vanishing depolarization field within the UFF. In this case, the UFF as thin as three unit cells have been reported to sustain stable polarization patterns in out-of-plane direction [142]. However, the case is different in realistic electrodes [143,144], where the screening charges distribute over a small but finite region in the metals (see Figure 2c). The screening charges in the electrodes are displaced from the interfaces and the compensation is incomplete. Therefore, these spatial charge distributions in electrodes lead to a voltage drop (see Figure 2d,e), which combines with the nonuniform polarization contributing to a nonzero depolarization field [125,132,145]:

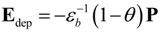
(14)
in a film under short-circuit (SC) boundary conditions, where 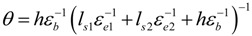 under the assumption of homogeneous polarization state. Here *h* is the thickness of the film. *ɛ_e_*_1_, *ɛ_e_*_2_ and *l_s_*_1_, *l_s_*_2_ are the dielectric constants and screening lengths of electrodes 1 and 2, respectively. *ɛ_b_* is the background dielectric constants. The depolarization field is dependent on the screening ability of the electrodes and the thickness of the ferroelectric thin films. In UFFs, the question how effectively the strong depolarization field can be screened is central to the critical thickness for ferroelectricity [123,146,147,148,149]. The screening effect of the electrodes has also been reported to play vital roles in the formation of ferroelectric domains [127,150,151]. 

For asymmetric thin film systems, e.g., those with dissimilar top and bottom electrodes, where the electrostatic environments at the two film surfaces/interfaces are diffident, an additional built-in field is built up. Assuming the work function steps for ferroelectric/electrode-1 and ferroelectric/electrode-2 interfaces to be Δ*φ*_1_ and Δ*φ*_2_ at P = 0, the built-in electric field can be given by [122]

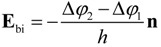
(15)
with **n** = **n**_1_ = − **n**_2_. **n**_1_ and **n**_2_ are unit vectors pointing from electrode-1 to electrode-2 and from electrode-2 to electrode-1. This additional built-in field is expected to cause a broken degeneracy of the states with out-of-plane polarization in antiparallel directions. As a consequence, effects such as imprint behavior, shifted hysteresis loops, and smearing of phase transition are expected [126,128,152]. As an important indication, a decrease and even vanishing of critical thickness for one polarization direction (and correspondingly an increase of critical thickness for the other polarization direction) would happen in asymmetric FTJs and FCs. These additional asymmetry-related features in asymmetric FTJs and FCs should have important implications on their applications. For example, the broken degeneracy of polarization states can lead to a loss of polarization bistability, which is crucial for applications based on polarization reversal such as memory. 

## 3. Simulation Methodologies

With the continuing advances in algorithms and computer hardware, computer-based calculation and simulation methods, being alternative to thermodynamic analysis, now are quite powerful in providing accurate insights into domain formation and evolution, thus playing central roles in promoting theoretically understanding of domain structures in UFFs. In this Section, we elaborate the basic aspects and developments of various numerical approaches for domain structure simulation, including first-principles calculations, MD simulation, MC simulation, effective Hamiltonian approach, and phase field method, with a particular attention to their technique features when tackling UFFs. The recent developments of the multiscale simulation scheme are presented, and the relative merits of each approach are also discussed.

### 3.1. First-Principles Calculations

First-principles calculations based on density-functional theory (DFT) calculate the properties of a system by the determination of ground state energy of the system within Born-Oppenheimer approximation. These calculations shed enormous light on understanding intrinsic properties of ferroelectrics with quantum mechanical accuracy within DFT. In particular, the origin of ferroelectricity can be understood from the viewpoint of electronic state. Moreover, the well establishment of the modern theory of polarization (as discussed in Section 2.1) makes it possible to compute the electric polarization and its derivatives including Born effective charges, dielectric and piezoelectric coefficients, *etc.*

First-principles calculations of the ground state structures of ferroelectric materials were first performed in the investigation of BaTiO_3_ and PbTiO_3_ by Cohen and Krakauer [72,73,74], and afterwards applied to a series of perovskite compounds [75], showing powerful capability in understanding and predicting ferroelectric behaviors. Then density-functional calculations were also employed to study ferroelectric solid solutions [153] and other ferroelectrics without perovskite structure like NaNO_2_ [154] LiNbO_3_ and LiTaO_3_ [155], *etc.* Most of these calculations are carried out with local density approximation (LDA) or other approximation forms on exchange-correlation functional. LDA calculations typically underestimate the lattice constants by about 1%, while calculations within generalized gradient approximations (GGA) yield a larger lattice value. In some cases, however, GGA leads to an overestimate of the lattice constants. The weighted density approximation (WDA) works as another density functional. Besides, Hartee-Fock (HF) method which is alternative to DFT has also been used in the investigation of ferroelectrics. The general aspects of the first-principles calculation method and its implementations can be found in Ref. [36,75,156] and references therein. 

We’d like to concentrate on the problems related to the first-principles modeling of ferroelectrics, especially methodologies for UFFs and domain structures here. For an UFF, where surface plays an important role via surface relaxation, reconstruction, and modification of the electrical and mechanical boundary conditions, it is natural to take an isolated planer slab as a fundamental geometry. This however, is applicable to only a few first-principles calculations resting essentially on HF method [157,158] because most of the density-functional implementations require three-dimensional periodicity. Generally, to satisfy it, slabs are treated with periodically repeated supercells, which contain a vacuum space separating the slabs. For this case, determining proper thickness of the vacuum regions is important, which should be thick enough to suppress the possible spurious interactions among the periodically repeated slabs. Other variables to be specified include the orientation and number of the atomic layers and the termination of the surfaces. Based on the periodic slabs, Cohen [159,160] calculated the surface relaxation of BaTiO_3_. Moreover, Padilla and Vanderbilt further performed calculations with fully relaxed atomic coordinates for both BaTiO_3_ [161] and SrTiO_3_ [162] surfaces. 

**Figure 3:**
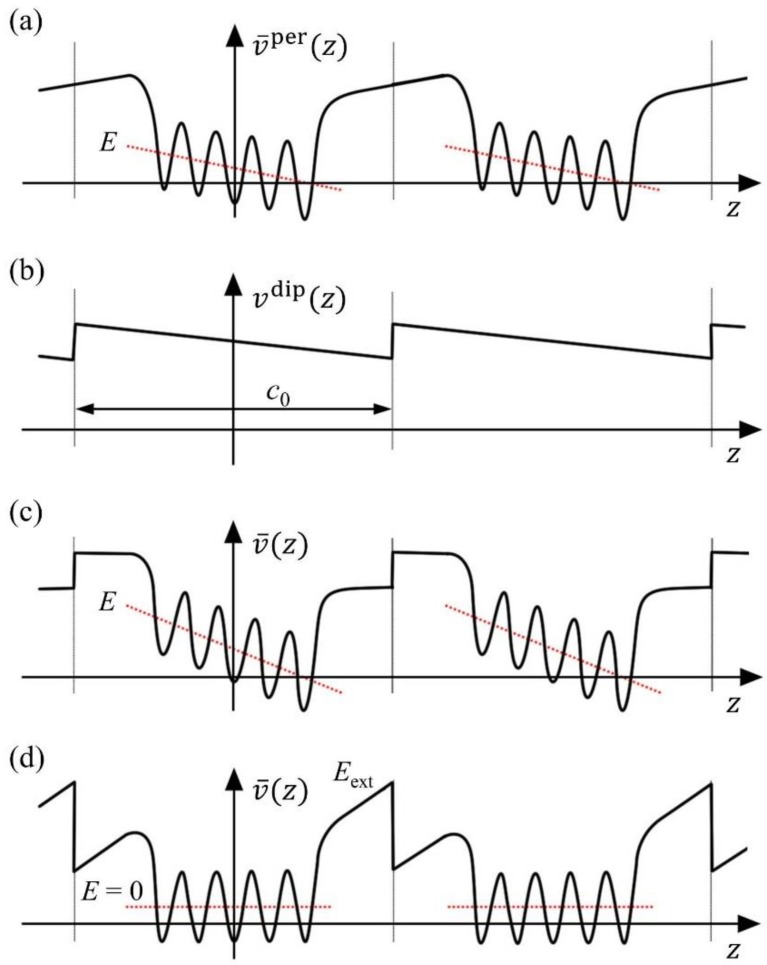
Schematic picture of the planar-averaged potential *v*(*z*) for periodically repeated slabs: (**a**) With periodic boundary conditions; (**b**) potential of the dipole layer; (**c**) dipole-corrected slabs with vanishing external electric field; and (**d**) dipole-corrected slabs with vanishing internal electric field. (Reproduced with permission from [164]; Copyright 2001 by the American Physical Society)

An important problem related to the geometry of periodically repeated slabs is the appearance of an artificial electric field along the normal direction in situations with non-vanishing electrostatic potential jump between two sides of the slab. Note that the imposition of periodic boundary conditions requires vanishing potential jump over the supercell, thus yielding a picture of the planner-averaged potential as shown in Figure 3a, which is exactly the effect of an applied electric field across the slab. This likely happens in slabs with nonzero out-of-plane polarization or nonequivalent surfaces. Although the undesirable field can be decreased by increasing the thickness of the vacuum in principle, it is usually expensive to do so. A dipole correction technique has been developed to overcome this problem [163,164], by introducing a dipole layer in the center of the vacuum region whose electrostatic potential is depicted in Figure 3b. Alternatively, introducing a dipole layer in the vacuum region can also serve as a useful method to apply a true external electric field to the surfaces. Meyer and Vanderbilt [164] have investigated the (001) surfaces of BaTiO_3_ and PbTiO_3_ in external electrical fields taking into account the dipole correction.

Ferroelectric capacitor (FC) can be investigated by replacing of the vacuum with metallic electrodes, forming a superlattice-like geometry. The metal layers usually need to be sufficiently thick to eliminate the interactions between copies of the thin film and make sure the application of short-circuit boundary condition. Junquera and Ghosez [146] discussed the ferroelectric instability of SrRuO_3_/BaTiO_3_/SrRuO_3_ capacitor based on a superlattice-like geometry. Similarly, superlattices of dielectric/ferroelectric or ferroelectric/ ferroelectric have been simulated [165,166,167]. To investigate FC structures, a multilayer geometry containing electrode/ferroelectric/electrode slabs separated by vacuum has been also used [148]. From an ideal point of view, this geometry can bring more physical surface/interface relaxation because no artificial constraint on the unit cell stains along the out-of-plane direction is imposed. Kolpak *et al.* [168] have discussed the benefits of both two FC geometries in their investigation of PbTiO_3_ thin films.

Both electrical and mechanical loads play important roles in properties of an UFF. In first-principles DFT calculations, external mechanical strain loads are mimicked by constraining some of the lattice constants of the supercell. For example, the effect of a substrate is simulated by fixing the in-plane lattice constants of the UFF to those of the bulk substrate. The application of the electrical boundary conditions has been a challenge. Nevertheless, great progress for different electrical boundary conditions has been achieved recently. Souza *et al.* [169] proposed a method to perform DFT calculations in finite electric field corresponding to adopting open-circuit (OC) boundary condition with a constant applied bias. A fixed polarization scheme was later developed by Dieguez *et al.* [170]. Besides, Stengel *et al.* [171] employed electric displacement as the fundamental variable in their electronic-structure calculations and suggested a constrained displacement method, which corresponds to a capacitor in OC boundary condition with a fixed value of the free charge on the plates.

**Figure 4:**
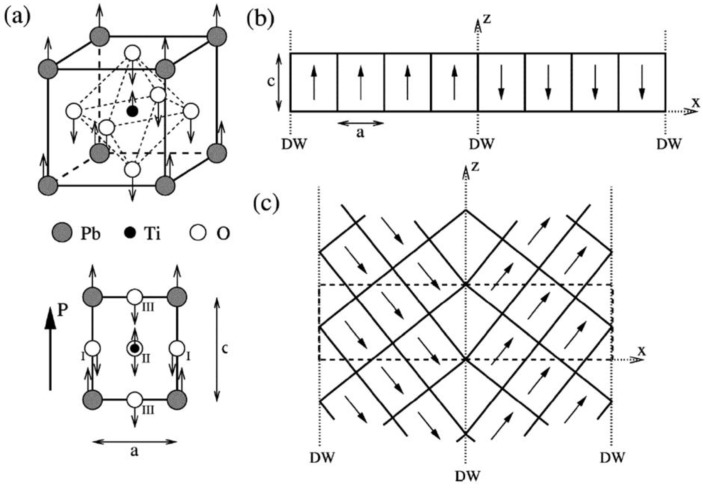
(**a**) Unit cell of the cubic perovskite structure and its projection along the [010] direction; arrows indicate atomic displacements in the ferroelectric tetragonal phase; (**b**,**c**) Supercell geometries containing eight perovskite primitive cells for (**b**) 180° and (**c**) 90° domain walls; atoms are omitted and only solid lines connecting the Pb atoms are drawn. Supercell boundaries are indicated by dashed lines. (Reproduced with permission from [173]; Copyright 2002 by the American Physical Society)

So far it is still challenged to apply first-principles calculations to simulate complex domain structure in UFF, due to the limited capability of large systems. Nevertheless, regular ferroelectric domain walls can be readily studied by first-principles DFT calculations based on the construction of appropriate supercells. For example, in the case of 180° domain walls in PbTiO_3_ [172,173], there exist two possible types of domain walls being Pb-centered (twinning on PbO plane) and Ti-centered (twinning on TiO_2_ plane), respectively. Supercells are usually built to consist of *N* × 1 × 1 perovskite unit cells arranged along the *x* direction and contain up and down domain as shown in Figure 4b. For 90° domain walls [173], as there is no sharp difference between a Pb-Ti-O or an O-O centered domain wall, thus we only need to perform the relaxation for any one of the two. The geometry of the supercell for 90° domain wall is depicted in Figure 4c, where the supercell is orthorhombic with dimensions of 
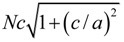
, *a*, and 
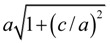
 in the *x*, *y*, and *z* directions, respectively. Furthermore, Lubk *et al.* [174] studied the 71°, 109° and 180° domain walls in BiFeO_3_. Recently, Shimada *et al.* [175] adopted first-principles calculations to elucidate the stability of 180° domain wall in ultrathin PbTiO_3_ films. The characteristics of such a domain wall in PbTiO_3_/SrTiO_3_ superlattices have also been investigated by Aguado-Puente and Junquera [176]. 

### 3.2. Molecular Dynamics Simulation

Molecular dynamics (MD) simulation is an atomistic level method treating atoms of the studied system as classical particles, whose motion can be described by the Newton’s second law or by Hamiltonian dynamics. Therefore, properties of interest can be determined by simulating the temporal evolution of the system governed by the equations of motions. Generally speaking, the basic idea of MD simulations is that the average behavior of a many-particles system can be determined by computing the natural time evolution of that system and averaging the quantity of interest over a sufficiently long time, known as time average. Note that MD simulation mimics the real time evolution of a system at finite temperature, which makes it quite superior when handling with dynamics problems. One can refer to Ref. [177] for very detailed information about the basic MD method.

#### 3.2.1. Potential Models for Ferroelectrics

The development of interatomic potentials exactly characterizing the behaviors of the investigated system is at the core of MD simulations. For ferroelectric materials, a successful potential model should capture the delicate balance between the short-range interactions beneficial for paraelectric phase and the long-range force favoring ferroelectric ordering. In particular, both the ionic and covalent interactions must be taken into account for ferroelectric oxides, which are mainly ionic material but exhibit covalency in some degree. The expressions of the interatomic potential should not be too complex, making it hard to be applied to simulate large systems. Moreover, the parameters of the potential should be fully specified by first-principles calculations.

The shell model, originally proposed by Dick and Overhauser [178], has been validated to be a successful interatomic potential model for ferroelectric materials (especially for ferroelectric oxides). In the shell model, each atom is modeled by a massive core linked to a massless shell by a spring. There are electrostatic interactions among cores and shells of different atoms, and short-range interactions between different shells. Therefore, the atomic polarizability can be easily described by the relative displacement between the core and shell of the atom. The covalency effects are taken into account when considering the atomic polarizability and determining the charges of the cores and shells [179].

First-principles based MD simulations with shell model for ferroelectrics were firstly developed by Stachiotti *et al.* in the investigation of KNbO_3_ [180]. They adopted an anisotropic core-shell interaction for oxygen to reflect the underlying anisotropy of the crystallographic environment of oxygen ions. The short-range interaction is chosen to be the Born-Mayer form except for the O-O interaction, where a Buckingham form is adopted by adding a Van der Waals term. The parameters were determined by first-principles total energy calculations. Tinte *et al.* [181] later adopted this model for BaTiO_3_ crystal and reproduced the full phase transition sequence of it. Nevertheless, for disordered systems, e.g., solid solutions and defected systems, it is hard to unambiguously characterize the crystallographic environment of any given ion; thereby, a second generation of shell model was then proposed [182,183]. In the second generation of potentials, the core-shell coupling was suggested to be anharmonic but isotropic, which both stabilizes the ferroelectric phases and prevents the very large local electric fields associated with the breaking of the cubic symmetry from making the core-shell displacements too large. 

Shell model MD simulations have also been applied to the investigation of ferroelectric materials including PbTiO_3_ [184,185,186], Pb(Mg,Nb) (PMN) [185,186], and PMN-PbTiO_3_ [186]. In these materials, considerable changes in the iconicity and covalency related to charge transfer are exhibited due to the large change of the bond length during the ferroelectric phase transitions. This, however, has not been taken into account in the traditional shell model. Actually, the feasibility of charge transfer being ignored above is believed to be critical in MD modeling of ferroelectrics especially in systems including surfaces and interfaces, where the atomic charges may vary significantly with changing local environment. One of the most important objectives of the next generation interatomic potentials is to introduce this additional feasibility to increase the accuracy of MD simulations. As an initiative attempt, Goddard and co-workers [187] have developed a ReaxFF approach to give a more flexible and realistic representation of the atomic interactions in BaTiO_3_. This has been reviewed in Ref. [179] and [188]. 

Another problem exists in MD modeling of ferroelectric phase transitions nowadays is the obvious underestimates of the transition temperatures. This error, however, is caused not by the shell model itself, but by the well-known underestimates of lattice constants in first-principles DFT calculations with LDA, thus exists in many other models whose parameters are specified based on a LDA database. Thankfully, the LDA error can be compensated by performing atomistic simulations under a negative pressure. Besides, there are also attempts to specify the parameters based on other density-functional approximation such as the Perdew-Burke-Ernzerhof GGA for solids, *i.e.*, PBEsol [189].

It is noteworthy that there are other potential models for ferroelectrics. Grinberg and co-workers have proposed an interatomic potential emphasizing the covalent interactions [190,191] based on the widely used bond-valence theory [192]. In this model, the bond valence of an ion is defined as a function of bond length. The bond-valence model has been used to the atomistic simulations of phase transitions and domain walls in PbTiO_3_ [193,194], as well as the structure and dynamic characteristics in PMN-PbTiO_3_ relaxor ferroelectric materials [195,196,197]. Dipolar potential model containing dipolar-dipolar interaction explicitly has been adopted in the MD simulations of PbTiO_3_ [198] and CaTiO_3_ [199]. 

#### 3.2.2. Techniques of Calculating Interatomic Forces

One practical issue faced with molecular dynamics simulations for ferroelectrics exists in the calculation of the interatomic forces based on the potential models discussed above, which is the most time-consuming part of MD simulation. 

Short-range interactions are usually truncated by a cutoff sphere of radius *r_c_* due to the rapid attenuation with distance. Nevertheless, the process of just calculating the interatomic distances *r_ij_* between the given atom and the others at each step is very time consuming, most of which are actually larger than *r_c_* and make no contributions. Verlet [200] suggested a method to avoid the useless calculations of *r_ij_* by constructing a neighbor list for each atom, commonly known as the Verlet list method. This method was then developed and discussed by Thompson [201] and O’shea [202]. Another efficient method is the cell list method [203]. The simulation region is divided into a lattice of small cells with a size larger than *r_c_*. Thus each atom only has interactions with atoms in the same and neighbor cells. The choice of calculating methods depends on the actual conditions of the system. The Verlet method is proven to be very efficient but storage consuming, while the cell list method is good at dealing with systems with large amounts of particles. A method with a combination of the two has been suggested by Auerbach *et al.* [204]. The efficiency of various methods has been discussed by Frenkel and Smit [205].

Calculation of the long-range forces (*i.e.*, electrostatic interactions) is a great challenge for computer simulations, since their long-range feature prevent a straightforward truncation. There have been many efforts to handle the electrostatic interactions more efficient and accurate. Ewald sum [206] is a classical method to sum the interactions between a given ion and all its periodic images. In the Ewald sum method, each point charge is assumed to be surrounded by a charge distribution of equal magnitude and different sign. Thus, the screened interactions are short-ranged and can be calculated in the real space. A compensating charge distribution is then imposed to cancel the screening charges. The interaction of these remaining charges can be readily summed in reciprocal space. Ewald sum is usually combined with particle mesh approaches, e.g. particle-particle/particle-mesh (PPPM) [207], particle mesh Ewald (PME) [208], and smooth particle mesh Ewald (SPME) [209], *etc.* Moreover, fast multipole method by Greengard and Rokhlin [210] has also been discussed [211,212]. However, note that the traditional Ewald summation requires periodicity in all three directions of the system, which is not the case for finite size ferroelectrics. Additional improvements [213] must be adopted when utilizing this method in ultrathin ferroelectric films.

There are also methods attempting to truncate the long-range interactions like the reaction filed method [214]. In partcular, Wolf and co-workers [215] have suggested that the net Coulomb potential in condensed ionic systems is rather short-ranged, thus can be truncated. This method was then discussed in detail [216]. It is noteworthy that this method can be directly applied to non-periodic systems, since there is no imposed periodic requirement like the Ewald sum. Therefore, it is widely applied to the molecular dynamics simulations for ferroelectrics, especially for the UFFs [139,140,217].

#### 3.2.3. Molecular Dynamics in Various Ensembles

Based on statistical mechanics, MD mimics realistic ferroelectric systems by simulating in various ensembles. Schemes at constant temperature (*i.e.*, the canonical (NVT) ensemble), and constant pressure (*i.e.*, the isobaric-isoenthalpic (NPH) ensemble), or more generally, constant applied stress, are usually adopted for UFFs. Many MD simulations are also performed in isothermal-isobaric (NTP) ensemble, taking into account the effects of fixing both the temperature and pressure simultaneously in the updates of the coordinates and velocities.

Woodcock [218] developed an isothermal MD in the year 1970. He suggested fixing the temperature of the system by rescaling the velocities of the particles at each time step. Although this method usually tends out to be very crude, and cannot generate states in the NVT ensemble, it is very simple and convenient. This constraint method was then improved by Berendsen *et al.* [219]. Andersen [220] proposed a stochastic method resulting in trajectories of NVT ensemble. In Andersen’s method, a heat bath is assumed with the desired temperature coupling to the system through stochastic impulsive forces that act occasionally on randomly selected particles, known as Andersen thermostat. Soon after, following Andersen’s scheme for constant pressures MD [220], Nosé [221,222] introduced a new degree of freedom to describe the interaction between the system and the heat bath, and expressed the Hamiltonian of the extended system as a sum of contributions from particles, and heat bath. Thus based on which, equations of motion were derived by some transformation. Nosé’s method was then implemented by Hoover [223] known as Nosé-Hoover thermostat. 

Techniques for MD at constant pressure developed at the same time with those at constant temperature discussed above, including constraint methods [219], Andersen’s extended system method [166], and Parrinello and Rahman’s method for varying cell shape systems [224,225,226], *etc.* In particular, Parrinello and Rahman discussed the constant pressure molecular dynamics as a special case of the MD at the applied stress using a new Lagrangian formulation [224]. In general, while perusing efficiency, the development of approaches for MD in various ensembles is becoming more and more rigorous and physics-dependent. A Nosé–Poincaré-Andersen approach [227,228] for NTP ensemble has been adopted in many MD simulations [229,230,231]. For this approach, a Poincaré time transformation has been applied to the traditional NTP Hamiltonian to make new Hamiltonian preserve time-reversal symmetry, which can take advantage of symplectic integration schemes, and can enhance stability for long-time simulations

### 3.3. Monte Carlo Simulation

Monte Carlo (MC) method works as another commonly used atomistic-level simulation approach for ferroelectrics. In MC method, properties of the studied system are determined by calculating the ensemble average based on the idea of Gibbs. To do so, it is central to MC technique to generate a set of states in phase space that are sampled from the complete sites in accordance with the desired probability density. Note that, different from MD simulation based on the classical mechanics, what generated here is only a virtual evolution trajectory satisfying the probability distribution of certain ensemble. This limits the application of MC in non-equilibrium systems, whereas this makes MC very smart for equilibrium problems. 

It is very convenient to combine MC simulation with various models including Ising model, thermodynamics free energy, and effective Hamiltonian approach, since it requires only calculations of the changes in the total energy with trial moves of the configuration to find the equilibrium state. Primitive MC simulations of ferroelectric domain structures are mainly based on the Ising model [232,233] and the Q-state Potts model [234,235], which is a generalization of the Ising model with Q metastable states and recovers to it as Q = 2 [236]. For ferroelectric thin films, the four-state Potts model was usually employed [237,238]. In this model, a ferroelectric thin film can be described by a two-dimensional array of cells denoted as *N_x_* × *N_z_* with *N_x_* and *N_z_* being the number of cells along *z* (longitudinal) and *x* (transverse) directions. Each cell is related to a dipole, and the orientation of the dipole is represented by a state variable called pseudo-spin taking one of the four possible states. There are interactions between neighboring pseudo-spins, and the total energy of the system is described by the Hamiltonian of Potts formalism [236].

In the year 2000, Potter *et al.* [239] developed a two-dimensional lattice-based MC simulation technique for the prediction of ferroelectric domain configurations. They utilizes a Hamiltonian for the total energy based upon electrostatic terms involving dipole–dipole interactions, local polarization gradients, and the influence of applied electric fields. The elastic strain energy contribution was taken into account in their following investigation [240]. The final configuration is determined by the energy minimization of an ensemble of electric dipoles. This model was then employed to study the effect of dipole defects on ferroelectric switching behavior [241,242,243]. Liu and co-workers further considered the Landau potential and employed MC simulations based on Ginzburg-Landau free energy in their investigations on properties of relaxor ferroelectrics [244,245], ferroelectric dipole configurations [246] and domain growth [247]. In particular, Xue *et al.* [248] performed a MC simulation based on the full set of free energy terms on the size effects on ferroelectric domain structures. Recently, a great deal of work on domain structures in UFFs with MC method is based on the effective Hamiltonian approach, which we would talk about in the next section. 

MC simulations based on the models for ferroelectric domains above are usually performed via the Metropolis algorithm. Compared with other methods, it needs not to calculate the time-consuming time-dependent Ginzburg-Landau (TDGL) equation in phase field simulation or the interatomic forces in MD simulation. It is noteworthy that, although much attention has been attracted by many other striking methods nowadays, MC method is quite efficient and is a good complement for the study of ferroelectric domain structures in large systems.

### 3.4. Effective Hamiltonian Method

Established in the mid-1990s, effective Hamiltonian method has become a powerful atomistic level method for simulating domain structure in displacive-type ferroelectrics. It begins with the construction of a simple model Hamiltonian (*i.e.*, the effective Hamiltonian) containing only degrees of freedom that are essential to ferroelectric phase transition. Parameters of the model Hamiltonian can be fully specified by first-principles calculations. Thus based on the effective Hamiltonian in combination with numerical simulations, properties of ferroelectrics can be well studied. The effective Hamiltonian method has been successfully applied to the study of various properties of many ferroelectric materials, e.g., domain structures, phase transitions, and size effects, *etc.*

#### 3.4.1. Construction of the Effective Hamiltonian

The construction of the effective Hamiltonian is always a nontrivial process, which should be based on the lattice dynamics of the ferroelectric. Here we will not focus on the detailed construction of the effective Hamiltonian, but discuss physics of the basic form for various ferroelectric materials, aiming to provide readers an instructive introduction on this method. Readers can refer to the papers by Rabe and Waghmare [249,250,251,252] for theoretical discussions of this method in detail. Note that the energy surface of a ferroelectric can be expanded according to the local mode, a coordinate related to the soft mode, and the strain. The effective Hamiltonian generally contains five parts for a simple perovskite, e.g., PbTiO_3_ [252], BaTiO_3_ [253], and BaZrO_3_ [254] *etc.*, which can be written as:

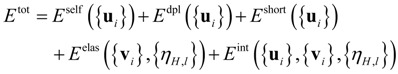
(16)
where **u***_i_* is the local soft-mode amplitude vector in unit cell *i*, and *η_i_* is the strain tensor. The first three terms are local mode self-energy, long-range dipole-dipole coupling, and short-range interaction between local modes, respectively. The fourth term is the elastic energy and the last term is the interaction between local modes and local strain. In literature, the model Hamiltonians for KNbO_3_ [255] and KTaO_3_ [256] have also been developed.

The effective Hamiltonian for alloy system PZT was developed by Belliche *et al.* [257]. They introduced a new variable *σ_j_* to characterize the atomic configuration of the alloy. Thus the total energy can be rewritten as:

*E*^tot^ = *E*^ave^ ({**u***_i_*} , {**v***_i_*} , {*η_H ,l_*}) + *E*^loc^ ({**u***_i_*} , {**v***_i_*} , {*η_H ,l_*} , {*σ_j_*}) (17)

Here the first term is the simple perovskite ABO_3_ energy due to virtual crystal approximation (VCA), and the second term is the local effect due to the random appearance of Zr and Ti at the B site. Then, similar Hamiltonians are applied to (Ba,Sr)TiO_3_ (BST) [258] and Ba(Zr,Ti)O_3_ (BZT) [259]. The effective Hamiltonian for PZT has been further improved by Belliche *et al.* [260] to include the antiferrodistortive (AFD) terms by a similar method.

The general form of effective Hamiltonian discussed above is not confined to bulk ferroelectrics. With appropriately treating those parts of Hamiltonian that are intimately related to the surfaces, various ferroelectric systems, including zero-, one-, two- and three-dimensionally periodic systems can be well described. Specifically, several aspects need to be considered when modeling UFF systems. No periodic boundary conditions should be imposed along the out-of-plane direction. Thus the expression of the dipole-dipole interaction is quite different from that in Equation (16). Some useful techniques should be adopted to handle the long-range dipole-dipole interaction in a two-dimensional system as discussed in Section 3.2. It is noteworthy that Naumov and Fu [261] have proposed a new method to deal with the dipole-dipole interaction in partially periodic systems recently. This method leads to a simpler expression based on the Green’s function and is widely used in the effective Hamiltonian method now. Ponomareva *et al.* [262] suggested that the depolarizing energy in a ferroelectric film can be calculated as the difference between dipole-dipole energies of OC electrical boundary conditions and SC electrical boundary conditions. And the latter one is exactly the same as the one described by its bulk counterparts.

Moreover, the intrinsic surface effects in an UFF have been discussed by Almahmoud *et al.* [141]. They proposed a surface energy term to mimic how the existence of free surfaces affects the dipoles and strains near them. Nevertheless, the interfacial effects are ignored by simply assuming the substrate to be inert. Despite this, the method was adopted in many following investigations [263,264,265]. No sophisticated scheme is available to our knowledge till now.

Therefore, the total energy of an ultra-thin ferroelectric film can be written as:

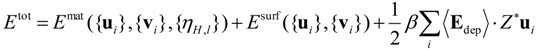
(18)
Here the first term is first-principle-derived effective Hamiltonian for bulk ferroelectrics with the dipole-dipole interaction modified as discussed above. The second term represents the interaction between the top surface and the vacuum. The effect of screening of the depolarization field is described in the third term via a factor *β*, where *β* = 0 corresponds to the ideal OC condition and *β* = 1 corresponds to the ideal SC condition. 〈**E**_dep_〉 is the maximum depolarization field inside the film. 

#### 3.4.2. Numerical Implement

It is convenient to combine the effective Hamiltonian with MC simulation, since it requires only calculations of the changes in the total energy with trial moves of the configuration to find the equilibrium state. The Metropolis algorithm MC simulation has been used to solve the effective Hamiltonian at the very beginning [253]. In the MC scheme, a large amount of MC sweeps are first preformed to equilibrate the system, and then another large amount of sweeps are used to get the statistical average. The simulations are usually quasi-static cooling-down process and the temperature is decreased in small steps. Moreover, besides the classical MC, the path-integral quantum MC method [256,266] has also been used to combine with the effective Hamiltonian in order to consider the zero-point motion of ions. 

MD simulation is another important method to simulate the systems described by effective Hamiltonian model. MD simulation has unique advantages of handling time-dependent problems, e.g., the domain evolution under an alternating electric field. However, implement of the MD simulation is more complicated compared with MC simulation. The effective Hamiltonian should be recast to include the kinetic energy terms at first. Besides, it is more computationally demanding to perform MD simulation than MC simulation based on the model Hamiltonian. Waghmare *et al.* [267] in the year 2003 developed a technique to increase the efficiency of MD simulation, which has been used in latter investigations [229,230,231]. In particular, they suggested calculating the interactions and inhomogeneous strain in reciprocal space based on fast Fourier transform (FFT). Furthermore, MD simulations with the effective Hamiltonian have been performed at constant temperature or constant pressure using the Nose-Poincare Method, which is symplectic as discussed above. Recently, MD simulations based on effective Hamiltonian method have been adopted by more and more researchers. A detailed discussion about it can be found in Ref. [230].

### 3.5. Phase Field Method

During the past three decades, phase field method has been demonstrated as a powerful approach to simulate the microstructure evolution problems of a wide spectrum of materials under various conditions (see Ref. [268] and the references therein). Phase field method for domain structure in ferroelectrics is generally based on the Landau-type thermodynamic model as discussed in Section 2.2. The evolution of domain structure of a ferroelectric system is simulated by solving the temporal dependence of an order parameter field, which is usually chosen as the spontaneous polarization **P** = (*P*_1_, *P*_2_, *P*_3_). The evolution of the polarization is governed by the time-dependent Ginzburg-Landau (TDGL) equation. 

#### 3.5.1. Free Energy

Based on the phenomenological theory, the free energy of a ferroelectric system should be generally constructed under the thermodynamic framework, with the material parameters being correctly defined for a given thermodynamic process. Taking a paraelectric crystal in the absence of surfaces and applied fields (*i.e.*, electric, magnetic or mechanical, *etc.*) as the thermodynamic reference, the free energy of a ferroelectric system can be written as the sum of the following components, *i.e.*,

*F* = ∫_*V*_(*f*_Land_ + *f*_elas_ + *f*_grad_ + *f*_elec_)*dV* + ∫_*S*_*f*_surf_*dS* (19)

where *f*_Land_, *f*_elas_, *f*_grad_, *f*_elec_ and *f*_surf_ are densities of the Landau free energy, elastic energy, gradient energy, electrostatic energy and surface energy, respectively. *V* and *S* are the occupied space and the surface of the ferroelectric. In Equation (19), the Landau free energy density *f*_Land_(*P_i_*) describes the bulk property of the ferroelectric, and is generally expressed as a polynomial expansion of the polarization (see, e.g., Equation (5) for the case of one component of polarization), with the expansion coefficients determined by fitting to the experiment data or first-principles calculations. The elastic energy density *f*_elas_(*σ_ij_*,*P_i_*), or *f*_elas_(*ε_ij_*,*P_i_*), describes the self-effect of elastic field (stress or strain) and its coupling with the polarization. The spatial polarization variation also contributes a gradient energy to the total free energy, whose density is a function of the polarization gradient, *i.e.*, *f*_grad_(*P_i_*_,*j*_). If there are surfaces of the ferroelectric, an additional surface energy density *f*_surf_(*P_i_*) is necessary to force a polarization relaxation nearby the surface. Moreover, to describe the effect of applied electric field and the depolarization field, an electric energy density *f*_elec_(*E_i_*,*P_i_*) is introduced. An explicated form of free energy for a ferroelectric thin film can be found somewhere else [27].

#### 3.5.2. Evolution Equation

The temporal evolution of the polarization field, which characterizing the evolution of the domain structure, is driven by the decrease of the total free energy. Specifically, it is a nonequilibrium process and can be phenomenologically described by the TDGL equation [269], *i.e.*,

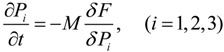
(20)
where *t* denotes the time; and *M* is the kinetic coefficient related to the domain wall mobility. 

Simulating the domain evolution is reduced to finding solutions to the TDGL equation (20). To do so, many different numerical methods can be implemented. Among them, the simplest one is the finite difference method employing a second-order central difference for the space derivative and a forward difference (*i.e.*, the simple forward Euler method or Runge-Kutta method when desiring higher accuracy in time) at time. It is noteworthy that in this scheme, the time step and spatial grid size have to be small enough to maintain the stability of the numerical solutions, yielding serious limitation on both the time and length scales to be simulated practically. Chen and co-workers [270] have developed an accurate and efficient semi-implicit Fourier-spectral method. They suggested transforming the partial differential Equation (20) into a sequence of ordinary differential equations in the reciprocal space using a Fourier-spectral approximation, and then solving them with a semi-implicit scheme instead of the forward Euler method. Despite the limitation of requiring periodic boundary condition, this method has been widely employed in phase field simulations of domain structures in ferroelectric systems such as UFFs [27,271].

#### 3.5.3. Numerical Methods of Calculating Electromechanical Fields

The mechanical field and electric field, playing crucial roles in behaviors of ferroelectric domain structures, are usually treated in an adiabatic way during the polarization evolution in the phase field simulation, under the assumption that mechanical and electric equilibrium can be instantaneously reached once the spontaneous polarization distribution is set down. Under this approximation, the mechanical field and electric field are determined by the mechanical and electrostatic equilibrium equations at a given local polarization distribution. 

To solve the mechanical equilibrium equation, the mechanical boundary condition in the UFFs should be specified. Following the microscopic elastic theory of Khachaturyan, the local strain can be represented as the sum of homogeneous strain and inhomogeneous strain. The homogeneous strain is the uniform macroscopic strain, while the inhomogeneous strain is chosen such that it yields no macroscopic deformation. In particular, for an UFF grown on a substrate, with the top surface being stress free and the interface between an UFF and substrate being mechanically coherent, the in-plane parts of homogeneous strains are controlled the misfit strains. Meanwhile, the other three homogeneous components meet the clamping condition. Specifically, the inhomogeneous strain distribution is determined by the mechanical equilibrium equation and the boundary condition equations with the homogenous strains subtracted. Due to the periodicity along the in-plane direction, numerical methods exploiting the efficient fast Fourier transform (FFT) technique are always advantaged over the real space methods such as finite element method (FEM), although the latter is powerful to calculate the equilibrium equations with arbitrary boundary conditions [272,273]. In the literature, Chen and his co-workers have developed a two-step scheme to calculate the inhomogeneous stress field in an UFF [271,274]. The equilibrium electric field can be solved in a similar way [150,275]. 

### 3.6. Multiscale Simulation

As mentioned in the previous sections, domain structures in an UFF can be studied by various computational methods, including first-principles DFT calculations, atomic level simulations (MD, MC and effective Hamiltonian method, *etc.*), and continuum methods (e.g., phase field method and analytic models), with their research scale ranging from atomic scale all way up to macroscale. Each type of these approaches has its relative merits and features. Specifically, first-principles DFT calculations determine properties of a ferroelectric system based on quantum mechanics without requiring any experimental input, and thus are regarded as the most rigorous and accurate method. First-principles calculations play central roles in our understanding origin of ferroelectric behaviors and have been employed to investigate ferroelectric nanostructures [24,25,276], effects of defects [277,278], or even domain walls [172,173,174,175,176] with the rapid development of the computer hardware. Nevertheless, the high demand of the computational power by first-principles calculations yields a serious limitation on the size of the system affordable to be only hundreds of atoms at zero temperature. At a result, issues like formation and dynamics of the ferroelectric domain structures at finite temperature are beyond the scope of these calculations. 

Probing these issues requires more computational feasible methods. MD simulations are capable of dealing with larger systems at finite temperature consisting of up to millions of atoms due to the simplification in describing interatomic interactions. Such an atomistic approach has the advantage of investigating dynamics and temperature effects [279,280,281]. However, weakness in transferability and accuracy of interatomic potential models available nowadays significantly hinders its application. Effective Hamiltonian method examines ferroelectrics based on the effective Hamiltonian containing only a few degrees of freedom closely related to ferroelectric phase transitions, which yields a quite simplified physical picture. This, however, requires a priori understanding of the critical degrees of freedom needed to characterize the system faithfully, and gives rise to question about effects of the ignored degrees of freedom at some particular conditions. Besides, both effective Hamiltonian method and MD simulation have similar limits on the time- and length scales that can be modeled practically. 

Phase field method is a very powerful method which can be applicable to a wide range of systems with quite large scales. Based on the phenomenological theory, parameters of the free energy nowadays are mainly determined by experiments [282,283,284]. It is noteworthy that the parameters measured under certain conditions may not be applicable to other conditions. Besides, the kinetic coefficients of the TDGL equation in phase field simulation are just assumed to be a constant to make sure the convergence of the numerical calculations, which leads to a fictitious temporal evolution. Therefore, the reliability of the phase field simulation needs to be improved. 

Note that fully investigating the domain structures requires combining computational methods of different scale. Therefore, a multiscale approach taking into account both the accuracy and large time- and length scale is expected. Unfortunately, a general framework of the multiscale scheme is still lacking. Only some of the scales are linked so far. We would like to take some representative examples here.

Results from first-principles DFT calculations can be transferred to serve as input parameters for larger scale methods. Actually, this is exactly what we have done in atomistic simulations, where the interatomic potential and effective Hamiltonian are all fully specified by first-principles calculations. Besides, parameters of the Landau-Devonshire free energy can be extracted from first-principles DFT calculations. For example, Duan *et al.* [138] made an attempt to link the density-functional results to phenomenological theory of an UFF to determine the extrapolation length of the system. Later, Gerra and co-workers [122,123] developed a multiscale scheme combining phenomenological modeling and first-principles calculations in their investigation of the size effect problem in ferroelectric capacitors. 

First-principle-based atomistic methods including MD simulations and effective Hamiltonian approach are promising to serve as a bridge between the electronic structure calculation and the thermodynamic calculations. Volker *et al.* [285,286] proposed a scheme to transfer results from first-principles calculations and atomistic shell-model simulation to a phase field model as an input for thermodynamic calculations. Kumar and Waghmare [287] present a method based on combination of constrained polarization molecular dynamics and thermodynamic integration to determine the free-energy landscape relevant to structural phase transitions and related phenomena in ferroelectric materials, bridging the gap between first-principles calculations and phenomenological Landau-type theories. Furthermore, MD simulations, as they have the advantage of tracing the intrinsic dynamics of polarization reversal and domain evolution [279,280,281], are expected to play central roles in determining the true kinetic coefficients of the TDGL equation employed in phase field simulations. To do so, much more attention is badly needed in future.

## 4. Results of Domain Structures in UFFs

In this Section, we review recent theoretical studies on some important issues of domain structures in UFFs. During the past decades, thermodynamic analytic approach has been widely applied in capturing the behaviors of domain structure in UFFs. Meanwhile, computer-based simulation and calculation and methods have also been rapidly developed to produce very detailed information relevant to ferroelectric domain structures, which enables us to trace realistic domain structures in UFFs corresponding to various conditions and promotes breakthroughs in some important issues together with thermodynamic analytic approach. As a consequence, the effects of interfacial electrostatics, boundary conditions and external loads on domain structures in UFFs have been intensively investigated during the past years, with the discovery of abundant novel and controllable properties related to the domain structures. In the following, rather than giving a complete review of recent theoretical results of the domain structures in UFFs, we would like to concentrate on some representative issues to provide insights into this rapidly developing field, with an emphasis on the effects of interfacial electrostatics, boundary conditions and external loads.

### 4.1. Effects of Surface, Interface, and Electrostatic Boundary Conditions

As already pointed out in Section 2.3 and Section 2.4, the polarization discontinuity at the surface or interface of an UFF would cause surface charges, which give rise to a depolarizing field. Such an electrostatic field plays a dominant role in formation of domain structures. Generally speaking, the surface charges can be compensated by electrodes, bulk free carriers and ionic adsorption from the environment, or can be decreased by polarization rotation near the surface. Moreover, domains in an UFF can also be influenced by the surface relaxation and interface chemistry related to the termination of the free surface and the chemical bonding between the UFF and the electrode at the interface, respectively. In literature, the stability of the different domains in UFFs with taking into account these effects is an active research issue, since maintaining ferroelectric ordering is the premise of most potential applications of an UFF. In this part, we first make a survey on recent findings on stability of ferroelectricity and polarization, and then turn to the domain structures in UFFs.

#### 4.1.1. Stability of Ferroelectricity and Polarization in UFFs

In the case of ideal SC boundary conditions where the surface charges are fully compensated, Ghosez and Rabe [142] reported that a stress-free symmetric TiO_2_-terminated (001) PbTiO_3_ thin film with 3 unit-cells thick maintains an out-of-plane monodomain. Real metallic electrodes, however, have only limited screening capability due to the finite screening length as discussed above, leading to a remaining depolarizing field. A first-principles study on realistic ferroelectric/electrode interface was first carried out by Junquera and Ghosez [146] within LDA. They predicted that BaTiO_3_ thin film between two metallic SrRuO_3_ electrodes in SC conditions loses ferroelectricity below a critical thickness of about six unit-cells. Then similar investigations on PbTiO_3_ FCs predicted a critical thickness ranging from 2 unit-cells to 7 unit-cells [147,148,149]. The inconsistent results of different studies mainly come from the difference in the density functional employed in these first-principles DFT calculations. For asymmetric FCs (AFCs), due to the difference between the work functions of the two ferroelectric/electrode interfaces, a built-in electric field develops, which is expected to cause two non-degenerated polarization states. Umeno *el al*. [288] investigated a Pt/PbTiO_3_/SrRuO_3_ AFC and found that the asymmetric combination of the electrodes enhances the ferroelectric polarization pointing from SrRuO_3_ to Pt while the opposite polarization becomes less stable. Furthermore, Gerra and co-workers [122,123] developed a multiscale scheme combining phenomenology and first-principles calculations to analyze the size effect on ferroelectric behaviors of FC and AFC. Their result on BaTiO_3_ FC with SrRuO_3_ electrodes is consistent with the assumption in the seminal work by Junquera and Ghosez, emphasizing the dominant role of the depolarizing field other than local interface chemistry in determining the critical thickness for ferroelectricity. This however, is not always true. In particular, Duan *et al.* [138] reported that a strong bonding at the ferroelectric/electrode interfaces can destroy the bulk tetragonal soft mode, favoring a creation of an interfacial domain wall in their first-principles study of KNbO_3_ FC. Recently, Stengel *et al.* [289] further confirmed the crucial effect of local chemical environment in UFFs, which is predicted to profoundly enhance the ferroelectricity in the case of interfaces formed between AO-terminated perovskites and simple metals. They then developed a comprehensive methodological framework via constrained displacement field first-principles DFT calculations for the computation and analysis of FC with realistic electrodes by making a rigorous separation between the interface and bulk contributions [290]. Based on a phenomenological modeling, Chen *et al.* [127,128] recently predicted that the misfit strain-temperature phase diagram of FCs and AFCs can be significantly shifted by changing the depolarizing field and built-in field respectively, which indicates promising controllability of domain structures by adjusting electrodes.

Surface bound charges can also be compensated by ionic adsorption. Recent investigations have shown that ferroelectric stability in an UFF without a covering electrode layer is extremely sensitive to the surface chemical environment. Molecules including O_2_, H_2_O, CO_2_, *etc.*, have been reported to have significant influences. In particular, Fong *et al.* [291] investigated epitaxial PbTiO_3_ thin films on conductive substrates with top surfaces exposed to a controlled vapor environment and found that the monodomain state in an UFF as thin as 3 unit-cells can be significantly stabilized by the surface charge passivation by OH adsorption. Wang *et al.* [292] further revealed that the variation in oxygen chemical potential at the surface plays a role of applied voltage, and by which the polarization of an UFF can be reversibly switched. Shin *et al.* [293] investigated the ultrathin BaTiO_3_ films exposed to water molecules, and concluded that small water vapor exposures do not affect surface structure or polarization, while large exposures result in surface hydroxylation and rippling, formation of surface oxygen vacancies, and reversal of the polarization direction. Nevertheless, all these mentioned above are experimental results. So far, there are relatively lacking of theoretical investigations taking into account the exact mechanism of ionic adsorption to predict the domain stability in UFFs [294]. In their work, Stephenson and Highland established a thermodynamic model for the equilibrium and stability of polarization in UFFs subjected to a chemical environment, with the degree of ionic compensation determined by electrochemical equilibria. This model is found to be in qualitative agreement with experiments [295], and new features of UFFs under ionic compensation such as an intermediate nonpolar state between positive polar and negative polar phases have been revealed. 

In case of poor screening, ferroelectric ordering in an UFF are stabilized by breaking up the system into domains. Usually, 180° out-of-plane stripe domains with vanishing total out-of-plane polarization are adopted in such systems, as reported by Stachiotti and Fang *et al.* [163,296] They further predicted critical thickness of a free-standing BaTiO_3_ stress-free film to be thicker than 7 unit-cells using MD simulations. Furthermore, based on first-principles DFT calculations, Shimada *et al.* [175] studied an ultrathin PbTiO_3_ film and showed that the ferroelectric closure domains can emerge. Such a polydomain state can provide more effective screening of the depolarizing field with respect to the ideal 180° domain wall configuration and was found to be energetically favorable over the paraelectric state even in the film as thin as one unit-cell thick, which indicates no critical thickness exists. 

#### 4.1.2. Domain Morphology and Evolution in UFFs

**Figure 5:**
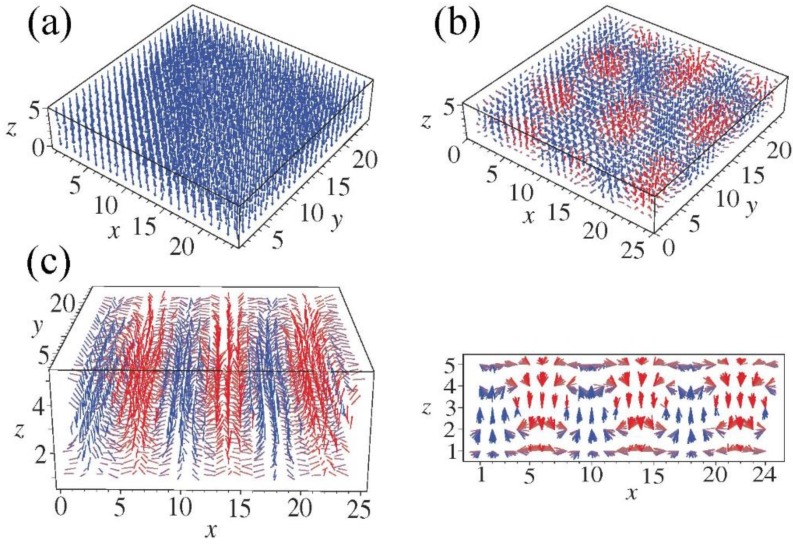
Three-dimensional polarization patterns in (001) PZT films having a 5 unit-cells thickness and under a compressive strain of 2.65% at 10 K. Panels (**a**), (**b**) and (**c**) correspond to the screening parameter of 94.5%, 87.7%, and 80.8%, respectively. The right part of (**c**) shows the projection of the three-dimensional picture into an *xz* plane. Red (blue) indicates local dipoles having a positive (negative) component along the *z* axis. 24 × 24 × 40 supercells are used. (Reprinted with permission from [151]; Copyright 2004 by the American Physical Society)

While most discussions on stability of ferroelectricity and polarization are based on first-principles DFT calculations and thermodynamic modeling, atomistic level simulations and phase field method enable us to deal with the morphology and evolution of ferroelectric domain structures in UFFs. Using a phase field method, Li *et al.* [150] studied the domain structures of PbTiO_3_ thin films under SC and OC boundary conditions and found that SC boundary condition promotes the formation of *c* domains (**P** = (0, 0, P_3_)), whereas OC boundary condition favors *a* domains (**P** = (P_1_, 0, 0)), indicating that the volume fraction of *a* domains and *c* domains can be adjusted by electrical boundary conditions. Kornev *et al.* [151] further depicted the evolution of the domains in a (001) PZT thin film under a compressive strain with different amount of surface charge screening as shown in Figure 5. 

**Figure 6:**
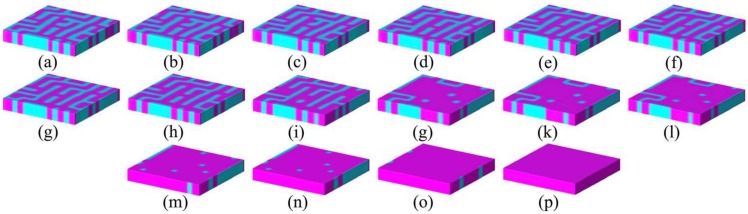
Domain evolution of the PbTiO_3_ thin film, *i.e.*, (**a**)–(**p**), controlled by the ionic charge density due to oxidizing atmosphere from 0.005 C/m^2^ to 0.0425 C/m^2^ with an interval 0.0025 C/m^2^. (Reprinted with permission from [297]. Copyright 2011, AIP Publishing LLC.)

Compared with ferroelectric thin films with electrodes, those with exposed surfaces usually exhibit especially rich set of domain patterns, since it is free for reconstruction and the bond charges can be compensated by a well-controlled chemical environment, providing insights into the nature of the bulk-to-film process. In particular, Ma *et al.* [297] investigated the evolutions of domain morphology in a PbTiO_3_ UFF subjected to the oxidizing atmosphere using a phase field method. They found that the chemical potential at the bare surface gives an analogous effect of applied voltage and can drive the domain wall movement with the continuous increase of oxygen ions density as depicted in Figure 6. Moreover, they predicted the critical surface ionic charge density which changes domain morphology of the UFF from a multi-domain to a monodomain state to be 0.0425 C/m^2^.

### 4.2. Effects of Strain

Ferroelectric materials usually possess strong polarization-strain coupling, thus properties of which can be greatly affected by macroscopic and microscopic strains. In thin films, strains including homogeneous strains and inhomogeneous strains can be introduced by film-substrate lattice misfit, defects, surface tension, and external mechanical loading, *etc.*, among which most are inevitable in preparation or processing stages. Simulations of strain effects not only contribute to the investigation of realistic ferroelectric materials, and more importantly, open an opportunity to manipulate the properties of UFFs by strains, which is known as strain engineering.

#### 4.2.1. The Role of Misfit Strain

**Figure 7:**
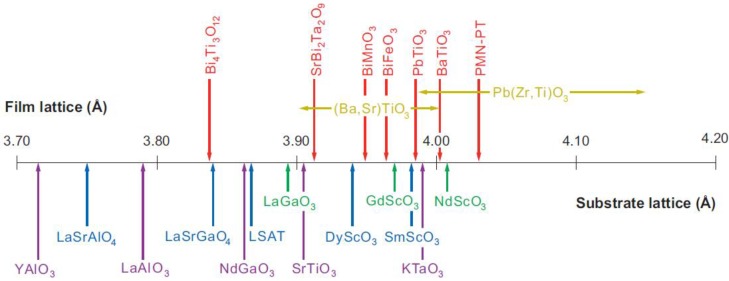
A number line showing the pseudotetragonal or pseudocubic *a*-axis lattice constants (in angstroms) of some ferroelectric perovskites of current interest (above the number line) and of some of the perovskite and perovskite-related substrates that are available commercially (below the number line). (Reprinted with permission from [110]; Copyright 2007 by Annual Reviews)

Misfit strain in a thin film originates from the differences in lattice parameters between the film and the corresponding substrate, thus from an ideal point of view it can be well controlled artificially by selecting different substrates. Generally, a coherent thin film can support considerable large strain up to several percent (with the record of 6.6% achieved in BiFeO_3_ thin films grown on YAlO_3_ [298]), which is far beyond the crack point of bulk crystal. Figure 7 adopted from [110] shows the pseudotetragonal or pseudocubic *a*-axis lattice constants of several typical ferroelectric perovskites and that of some popular substrates commercially available nowadays. Note that most substrates possess lattice parameters ranging from 3.8 to 4.0Å, implying easier implement of large compressive misfit strain experimentally for most materials listed in this figure. 

**Figure 8:**
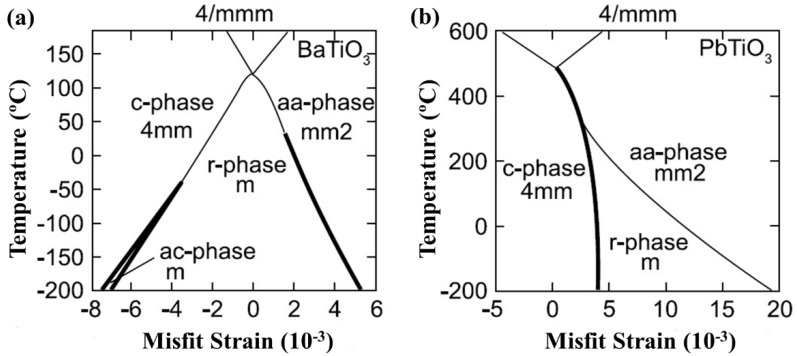
Misfit strain-temperature phase diagrams of (001)-oriented (**a**) BaTiO_3_ (Reprinted with permission from [18]; Copyright 1999 by Taylor & Francis) and (**b**) PbTiO_3_ (adopted from [17]) thin films calculated by thermodynamic analysis. (Reprinted with permission from [17]; Copyright 1998 by the American Physical Society).

Effects of in-plane strain (typically, misfit strain) together with the temperature effects on stability of ferroelectric phases and domain patterns in ferroelectric thin films, have been the focus of numerous theoretical articles. To graphically describe these effects, the so-called misfit strain-temperature phase diagram, showing ferroelectric phases or domain patterns as a function of misfit strain and temperature, has been constructed for many ferroelectric thin films. Common examples include BaTiO_3_, PbTiO_3_, PZT, (Ba,Sr)TiO_3_ (BST), as well as SrTiO_3_, an incipient ferroelectric in its bulk form and involves oxygen octahedral distortions. The pioneering theoretical works were based on thermodynamic analysis. By an assumption of single domain state, Pertsev *et al.* constructed phase diagrams of (001)-oriented BaTiO_3_ [18] and PbTiO_3_ [17] thin films (see Figure 8), where the equilibrium ferroelectric phases of a thin film at a given misfit strain and temperature is determined by comparing the energies of all possible ferroelectric phases as well as the parent paraelectric phase. These theoretical phase diagrams have notable features. Large compressive misfit strain favors *c* phase while *aa* phase (*P*_1_ = *P*_2_ ≠ 0, *P*_3_ = 0) is stabilized by large tensile misfit strain. In particular, for PZT thin film [20], the *r* phase (*P*_1_ = *P*_2_ ≠ 0, *P*_3_ ≠ 0) separating the *aa* phase and *c* phase at low temperature is found to widen with the increase of Zr content, with its center shifting from positive values of the misfit strain towards zero. The misfit strain-temperature phase diagram of SrTiO_3_ has also been developed based on thermodynamic analysis by Pertsev and co-workers [21]. They predicted misfit strain inducing ferroelectric phase in SrTiO_3_ thin film.

**Figure 9:**
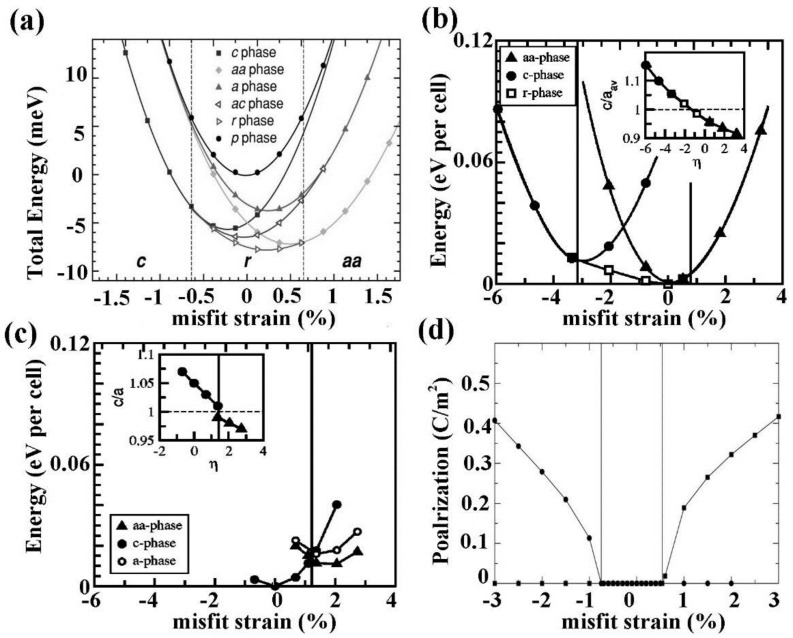
First-principles calculations of energies of all possible phases as a function of misfit strain in (**a**) BaTiO_3_; (Reprinted with permission from [299]; Copyright 2004 by the American Physical Society) (**b**) (PbTiO_3_)_1_(PbZrO_3_)_1_ superlattice; (Reprinted with permission from [300]; Copyright 2004 by the American Physical Society) and (**c**) PbTiO_3_ thin films; (Reprinted with permission from [300]; Copyright 2004 by the American Physical Society) and (**d**) polarization as a function of misfit strain in SrTiO_3_ thin films. (Reprinted with permission from [301]; Copyright 2005 by the American Physical Society)

First-principles calculations have also been directly employed to predict the equilibrium ferroelectric phase of epitaxial single domain UFFs at zero temperature. Based on this method, the *c*→*r*→*aa* phase sequence as a function of misfit strain at low temperatures predicted by thermodynamic analysis was further confirmed in BaTiO_3_ thin film by Diéguez *et al.* [299] and PZT thin film by Bungaro and Rabe [300] as shown in Figure 9a,b, respectively. However, first-principles calculations of PbTiO_3_ thin film showed that the intermediate *r* phase supposed to present at low temperature by thermodynamic analysis has been eliminated, and a mixture of *c* and *aa* phase is energy favorable instead, see Figure 9c. Moreover, Antons *et al.* [301] obtained the misfit strain tuning of polarization state in SrTiO_3_ thin films, see Figure 9d, which is in consistence with the results of thermodynamic analysis performed by Pertsev *et al.* [21].

**Figure 10:**
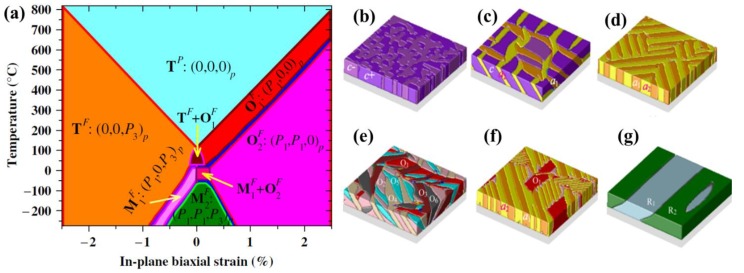
(**a**) Phase diagram of BaTiO_3_ films as a function of temperature and misfit strain, with **T***^P^* being tetragonal paraelectric, **T***^F^* being tetragonal ferroelectric, **O***^F^* being orthorhombic ferroelectric, and **M***^F^* being monoclinic ferroelectric. (**b**)–(**g**) Representative domain morphologies in BaTiO_3_ films at different temperature and misfit strain. Domain definitions and the corresponding polarization: *a*_1_: (*P*_1_,0,0); *a*_1_: (*P*_1_,0,0); *O*_1_: (*P*_1_, *P*_1_,0); *O*_2_: (*P*_1_, −*P*_1_,0); *O*_3_: (*P*_1_, ,0, *P*_3_); *O*_4_: (*P*_1_, ,0, −*P*_3_); *O*_5_: (*0*, *P*_1_, *P*_3_); *O*_5_: (*0*, *P*_1_, −*P*_3_); *R*_1_: (−*P*_1_, −*P*_1_, *P*_3_); *R*_2_: (*P*_1_, −*P*_1_, *P*_3_). (**b**) phase **T***^F^* at *T* = 25 °C and *e*_0_ = −1.0%; (**c**) phase **T***^F^* + 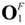 at *T* = 75 °C and *e*_0_ = 0.0; (**d**) phase 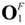 at *T* = 50 °C and *e*_0_ = 0.2%; (**e**) phase 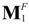 + 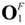 at *T* = −25 °C and *e*_0_ = 0.1%; (**f**) phase 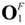 + 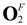 at *T* = 25°C and *e*_0_ = 0.25%; (**g**) phase 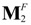 at *T* = −100 °C and *e*_0_ = 0.1%. (Reprinted with permission from [302]. Copyright 2006, AIP Publishing LLC.)

Note that the phase diagrams discussed above only applies for thin films in a single-domain state, which is usually energy unfavorable in practice. There were studies taking into account the multidomain structures based on thermodynamic analysis by assuming simply two-dimensional two domain states thin films [22]. Three-dimensional multidomain states in UFFs can be readily simulated based on phase field method and atomic level simulation methods. In particular, Li *et al.* [302] simulated the misfit strain effects on (001)-oriented BaTiO_3_ thin film under SC boundary condition using phase field method, and constructed a misfit strain-temperature phase diagram as shown in Figure 10. Notable features compared with the single-domain phase diagram of BaTiO_3_ include that there are several narrow regions with the coexistence of two or more domains, and when varying the substrate constraint from compressive to tensile near room temperature, the ultrathin film exhibits domain structure sequence from *c*-domains to *c*/*a*_1_/*a*_2_, then to *a*_1_/*a*_2_ twins, a mixture of *a*_1_/*a*_2_ and *O*_1_/*O*_2_ twins, and *O*_1_/*O*_2_ twins. Examples of domain structures from simulations corresponding to different phase in the phase diagram are shown in Figure 10b. Investigations on this issue based on the effective Hamiltonian approach were carried out by Lai *et al.* [303] with SC and near SC boundary condition and Yu *et al.* [304] in the case of OC boundary condition. In particular, Gui *et al.* [305] simulated the misfit strain-temperature phase diagram of epitaxial (110)-oriented BaTiO_3_ thin films and reported the existence of specific monoclinic and triclinic phases near room temperature.

**Figure 11:**
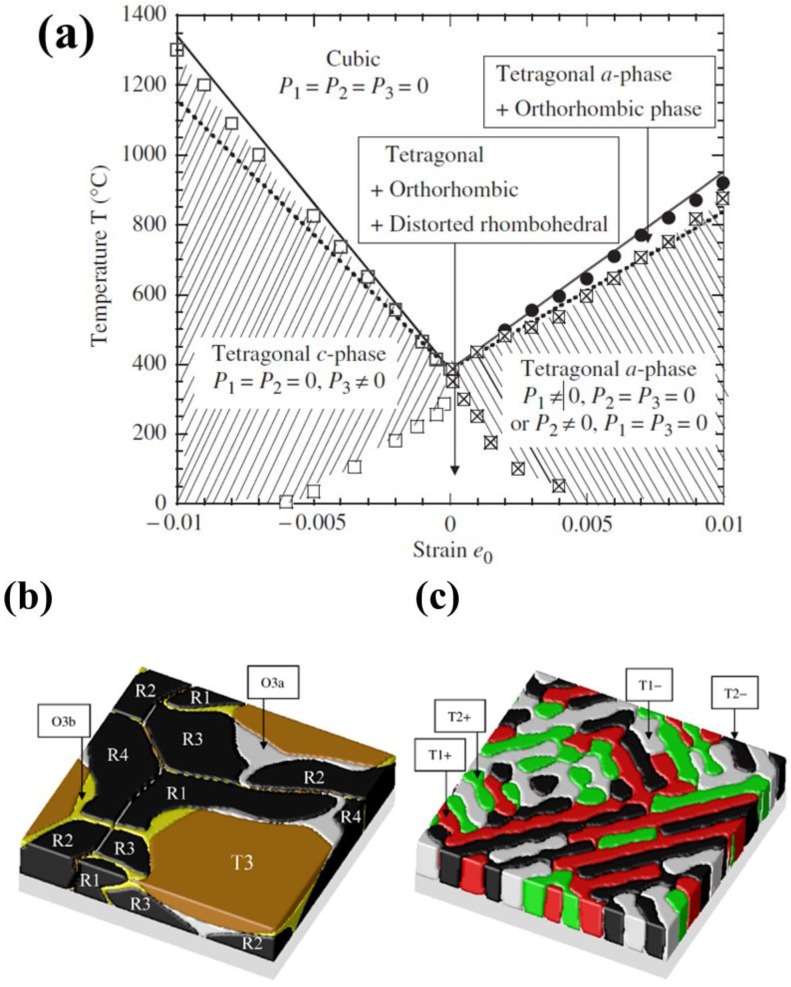
(**a**) Phase diagrams of PbZr_0.53_Ti_0.47_O_3_ epitaxial film and domain structures of the PbZr_0.53_Ti_0.47_O_3_ thin film at (**b**) *T* = 25 °C and *e*_0_ = −0.005; (**c**) *T* = 25 °C and *e*_0_ = 0.008 obtained from the phase field simulation (adopted from [310]). (Reprinted with permission from [310]. Copyright 2005, John Wiley and Sons).

**Figure 12:**
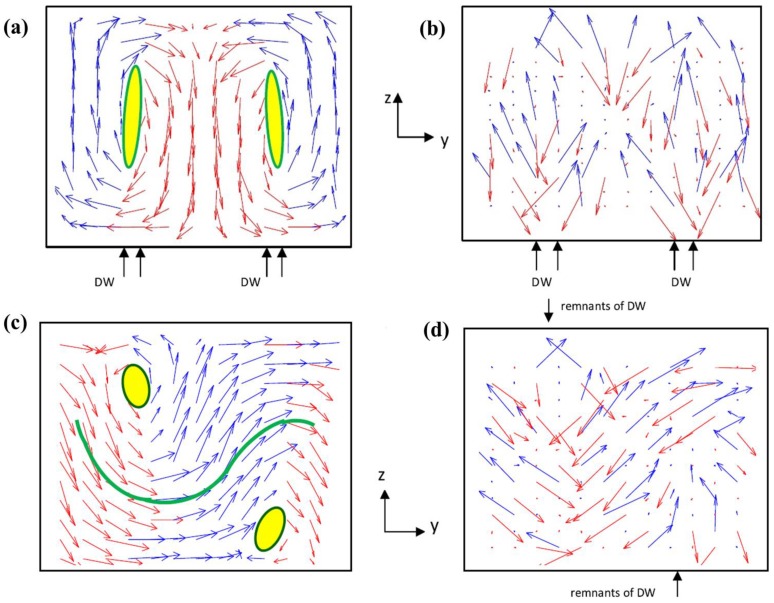
A 10K pattern of the (**a**) electric dipoles and (**b**) AFD vectors in a (*y*, *z*) plane for the *c^d^*[*R*(*c*)] state; and (**c**) electric dipoles and (**d**) AFD vectors in a (*y*, *z*) plane for the *aac^d^*[*R*(*ac*)] state of a 4.8nm-thick PbZr_0.52_Ti_0.48_O_3_ film under a compressive strain of −2%, as mimicked by a 12 × 12 × 12 supercell. (Reprinted with permission from [313]; Copyright 2011 by the American Physical Society)

Phase diagrams of PbTiO_3_ [124,274], SrTiO_3_ [306], BST [307,308], and PZT [309,310,311,312,313] have also been constructed based on the numerical approaches. In particular, for the solid solution PZT thin film, phase field simulations have shown that the tetragonal phase remains stable at a high mole fraction of Ti for compressive, tensile, and stress-free substrate constraint, while the orthorhombic phase is stable at low mole fraction of Ti for tensile constraint [309]. It is noteworthy that, misfit strain effect for (001)-oriented PZT thin films at the morphotropic phase boundary (MPB) composition, which exhibits strong electromechanical coupling, is of keen interest. Figure 11a shows a misfit strain-temperature phase diagrams for (001)-oriented PbZr_0.53_Ti_0.47_O_3_ thin films constructed by Choudhury *et al.* [310] based on phase field method, with two selected domain structures depicted in Figure 11b. According to the phase diagram in Figure 11, there are narrow regions where different domain states coexist, and large tensile misfit strain is found to favor tetragonal *a*_1_/*a*_2_-domain structure. Besides, a mixture of ferroelectric phases is reported to be stable at small strain here. Note that this is another difference from result based on thermodynamic analysis. The misfit strain-temperature phase diagram for (001)-oriented PbZr_0.52_Ti_0.48_O_3_ thin films under SC and OC boundary conditions was calculated by Sichuga *et al.* [312,313] using an effective Hamiltonian approach. Interestingly, two novel phases (the *c^d^*[*R*(*c*)] state and *aac^d^*[*R*(*ac*)] state as shown in Figure 12), both exhibiting dipolar nanodomains and oxygen octahedral tilting, are discovered in the latter case. This discovery provides a microscopic picture of the interplay between dipolar, antiferrodistortive, alloying, and strain degrees of freedom*.* Moreover, the domain morphologies in (001)-oriented PbZr_0.8_Ti_0.2_O_3_ and PbZr_0.2_Ti_0.8_O_3_ thin film have also been simulated by Li *et al.* [311] using phase field simulations.

It is noteworthy that previous discussions are focused on the isotropic misfit strain, while there are conditions anisotropic misfit strain can occur practically. For example, the misfit strain can be anisotropic when UFFs are grown on tetragonal substrates instead of cubic substrates. There have been articles concentrating on this issue based on thermodynamic analysis [112]. Using a phase field method, Sheng *et al.* [314,315] also investigated the effects of anisotropic strains on the domains structures of PbTiO_3_ and BaTiO_3_ thin films, resulting in fruitful misfit strain-misfit strain domain stability diagrams at certain temperatures. 

#### 4.2.2. The Role of Misfit Dislocations

Misfit dislocations, generated to release the strain energy arising from the lattice mismatch and or thermal expansion coefficient differences between the film and the underlying substrate, are commonly observed in UFFs when their thickness exceeds a critical value [316]. They play their roles in influencing domain structures by introducing an inhomogeneous strain field in addition to the reduced epitaxial strain. The effect of misfit dislocations on ferroelectric phase stability and domain formation in thin films was previously studied using the concept of effective misfit strain [317,318,319]. To take into account the local effects of dislocations on polarization distribution, Hu *et al.* [320] investigated the effects of interfacial dislocations in PbTiO_3_ UFFs subject to a substrate constraint using a phase-field method. With taking into account different oriented dislocation, they predicted that the presence of dislocation modifies locally the ferroelectric transition temperature and leads to the preferential formation of ferroelectric domains around misfit dislocations. Meanwhile, Alpay *et al.* [114] developed a thermodynamic model based on the stationary Landau-Devonshire equation to investigate the effect of the dislocation field on the spatial variation of the polarization field, and found that an interfacial dead layer of polarization exists due to the interfacial dislocations. Zheng *et al.* [321,322,323] further simulated the effects of interfacial dislocations on the domain structure of ferroelectric thin films based on a dynamic Ginzburg–Landau equation, with considering the effect of surface relaxation, which confirmed the existence of a dead layer near the film/substrate interface. Subsequently, they made systematical simulations to reveal the effects of misfit dislocations on properties of UFFs, including the polarization field, Curie temperature, dielectric constant, remnant polarization and coercive field, *etc.* Li *et al.* [324] further simulated the influence of the interfacial dislocations on the ferroelectric hysteresis loop employing phase field simulations. Moreover, the effect of threading dislocation [325], generally considered to be formed in the film by the glide of a dislocation half loop terminated at the surface, driven by the misfit stresses during film growth, has been also discussed. 

#### 4.2.3. Manipulating Domain Structures in UFFs by External Mechanical Loads

**Figure 13:**
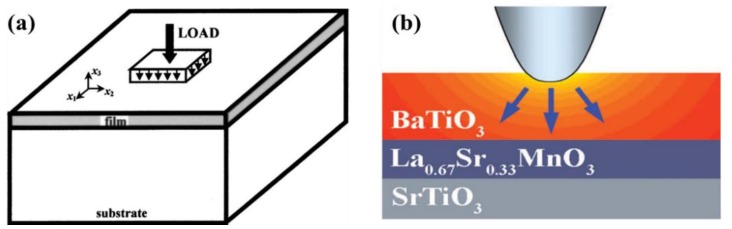
Schematic of (**a**) a uniform local loading (Reprinted with permission from [23]; Copyright 2002 by the American Physical Society) and (**b**) the strain gradient and associated flexoelectric field (arrows) induced by the atomic force microscopy (AFM) tip pushing on the surface of an ultrathin ferroelectric films (UFF). (Reprinted with permission from [329]; Copyright 2012 by AAAS).

**Figure 14:**
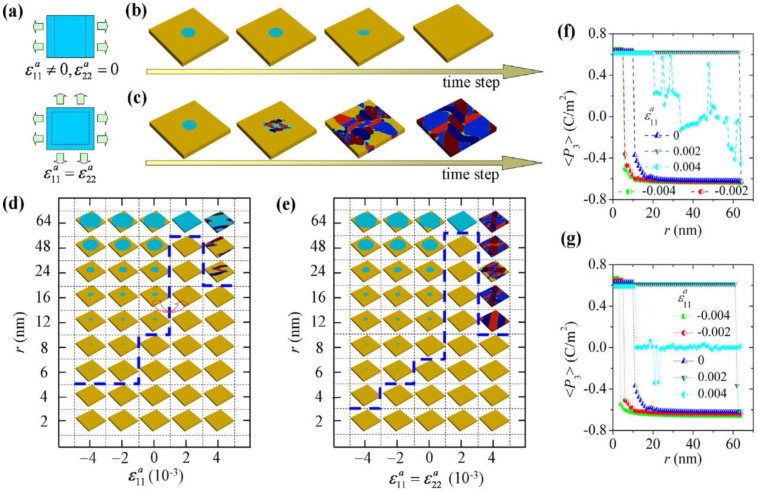
Control of domain stability by uniform strain on 128 nm × 128 nm × 8 nm simulation cells at room temperature. The cells are initially written with cylindrical domains with size *r* from 1 nm to 64 nm. (**a**) Schematics of a cell under biaxial strain and uniaxial strain. Domain evolution in a cell with cylindrical domain (*r* = 16 nm) under (**b**) 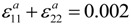 and (**c**) 
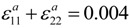
. Phase diagrams of equilibrium domain pattern in cell under (**d**) uniaxial strain and € biaxial strain. (**f**) and (**g**) the average polarization of the equilibrium domain patterns in *z*-direction, *i.e.*, <*P*_3_>, in the initial cylindrical domain region, for the two loading cases (Reprint with permission from [330]).

Considering the possible problems of electrical means nowadays (e.g., leakage, heat, dielectric breakdown and fatigue), and potential applications of ferroelectrics in non-electrical environments, effective strategies for processing ferroelectric domains by mechanical means would be meaningful. However, compared with the numerous articles being focused on utilizing film-substrate misfit strains to obtain novel ferroelectric domain structures in UFFs, attentions on manipulation of domain structure in UFFs by mechanical loads (*i.e.*, treating mechanical loads in a similar role of electric field) is surprisingly few. Using thermodynamic analysis, Emelyanov *et al.* [23] studied effects of uniform external mechanical forces acting on the upper surface of an epitaxial thin film grown on dissimilar cubic substrates. Figure 13a is a sketch of the situation. Investigation on effects of the uniform external mechanical loads on domain structures in UFFs with anisotropic misfit strains were carried out by Qiu and Jiang [113]. Note that driven by the repaid development of mechanical loading techniques such as the atomic force microscopy (AFM) (see Figure 13b) and integration of UFFs on compliant substrates, it is now becoming a trend to apply flexible mechanical loads to manipulate the domain structure of UFFs rather than just treating them as fixed constraints such as the misfit strain of rigid substrates [291,326,327,328,329]. More recently, Chen *et al.* [330] explored using mechanical means to control the stability of 180° cylindrical nanodomains in UFFs based on a phase field method. As indicated by the arrow in Figure 14, if a relatively small domain is written at compressive strain condition, by changing the strain condition to be less compressive, the domain becomes instable and backswitches, indicating an effect of mechanical erasing. Further simulation also shows that *a*/*b*-domains can be induced by large tensile strain, leading to the evolution of initial 180° cylindrical domain pattern into a complex domain pattern. Interestingly, results show that mechanical erasing can still be achieved in some cases even though *a*/*b*-domains were induced.

### 4.3. Effect of External Electrical Loads

Regulation of polarization and domain structures in UFFs by an electric field is straightforward and industrially feasible, thus is central to many ferroelectric devices nowadays. The depolarizing field governing the stripe dipole patterns can be distorted by a strong, global or localized applied electric filed, which can be either homogeneous or inhomogeneous. Therefore, based on this, novel formation and evolution path of ferroelectric domain structures in UFF may be induced, and this implies great potential for such devices. 

#### 4.3.1. Manipulating Domain Structures in UFFs by Homogeneous Electric Field

**Figure 15:**
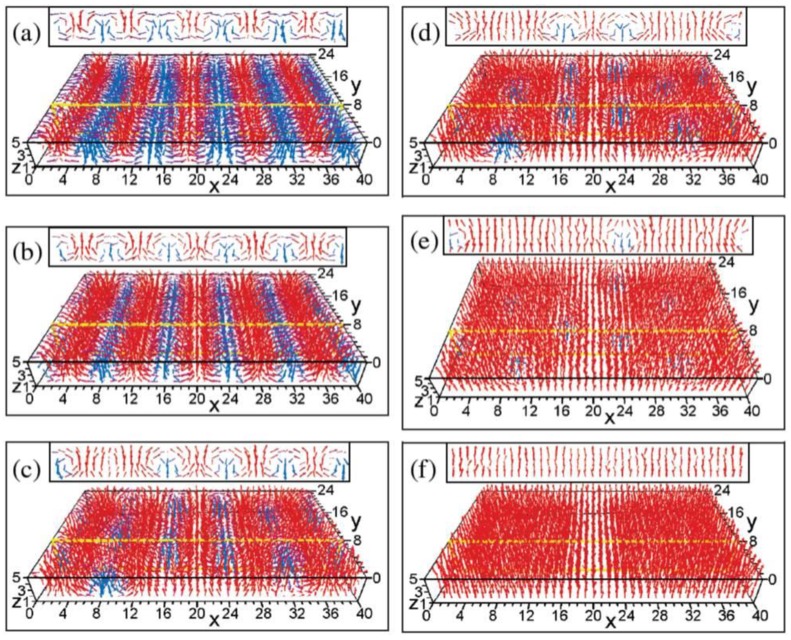
Domain structures in in (001) PZT films at 10 K under different electric field *E_z_*: stripe domains under (**a**) *E_z_* = 0 and (**b**) *E_z_* = 18 × 10^7^ V/m; bubble domains under (**c**) *E_z_* = 25 × 10^7^ V/m; (**d**) *E_z_* = 31 × 10^7^ V/m; and (**e**) *E_z_* = 43 × 10^7^ V/m; monodomain under (**f**) *E_z_* = 47 × 10^7^ V/m. The insets show the cross sections of a specific (*x*, *z*) plane (indicated by yellow lines). Red (blue) arrows characterize local dipoles having a positive (negative) component along the *z* axis. (Reprinted with permission from [331]; Copyright 2006 by the American Physical Society).

Similar to the case in ferromagnetic films, exotic dipole pattern of bubbles can be induced by a homogeneous electric field. Using a first-principle-based effective Hamiltonian, Lai *et al.* [331] focused on the electric field induced domain evolution in (001)-oriented PZT ultrathin films, as shown in Figure 15. 

The 20 Å-thick compressively strained film adopts stripe domain structures alternating along [100] direction with a periodicity of 8 unit-cells under zero external field, When an increasing *z*-direction electric field is applied, the film experiences a transformation of domain structure from stripe-type to nanobubble type and finally to a monodomain state. Similar transformation sequence was then obtained for (001)-oriented BaTiO_3_ ultrathin films with stripe domain structures alternating along [1,2,3,4,5,6,7,8,9,10] direction under vanishing external field [332]. Nevertheless, there are notable differences between the PZT and BaTiO_3_ ultrathin films. For the latter ones, before switching, Before switching directions, the dipoles antiparallel to the electric field decrease in magnitude at the very beginning which is not found in PZT. Others include the appearance of the zigzagged domain walls and the direction where the bubbles contract along. Furthermore, Sichuga and Bellaiche [333] investigated the situation of applying an electric field applied along the [111] direction for PZT UFFs. As the field influences simultaneously the in-plane and out-of-plane components, two new states, *i.e.*, the dipolar wave state and transitional state, are predicted to occur. Therefore, the domain structure of the PZT film under such a field application exhibits a transformation in a sequence of stripe state, dipolar wave state, nanobubble state, transitional state and finally monodomain state. 

**Figure 16:**
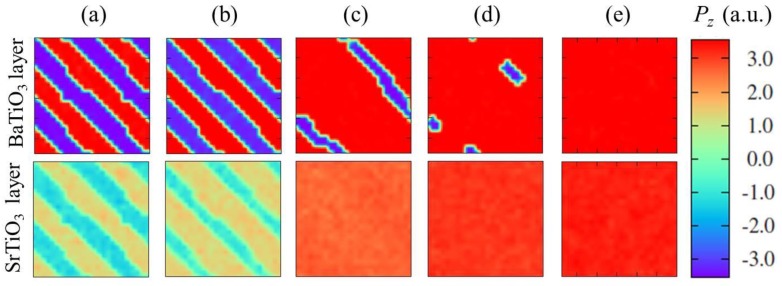
(001) Cross sections of dipole patterns in a BaTiO_3_ layer (top row) and SrTiO_3_ layer (bottom row) at *T* = 10 K, in the [BaTiO_3_]_10_/[SrTiO_3_]_10_ superlattice for different static electric fields: (**a**) *E* = 0.0 MV/cm; (**b**) *E*=2.65 MV/cm; (**c**) *E* = 3.0 MV/cm; (**d**) *E* = 4.5 MV/cm; and (**e**) *E* = 5.8 MV/cm. The color reflects the out-of-plane component of the electric-dipole moment, *i.e.*, *P_z_*. (Reprinted with permission from [279]; Copyright 2009 by the American Physical Society)

Artemev *et al.* [334] studied the domain structure in bilayer ferroelectric films. They found that a self-poled state can be produced in a bilayer film with one layer in a polydomain state and the other layer in a single-domain state at zero applied field. Increasing the applied field can lead to a single-domain state in the whole bilayer. The coupling between the domain structures of different layers has also been studied in superlattice structures. For ferroelectric BaTiO_3_/SrTiO_3_ superlattices, first-principles based calculations have shown that the domain pattern of the BaTiO_3_ layer and the SrTiO_3_ is similar at zero applied field. Interestingly, a difference in the domain pattern between the two layers is observed when the applied filed increases [279]. This implies a decoupling between BaTiO_3_ and SrTiO_3_ layers induced by electrostatic field, as shown in Figure 16.

#### 4.3.2. Manipulating Domain Structures in UFFs by Inhomogeneous Electric Field

There are many cases where we apply an inhomogeneous electric field rather than homogenous electric field to manipulate domain structures in UFFs. Compared with homogenous field, which usually requires sufficiently large parallel electrodes and thus occupies a large space, inhomogeneous field can be generated by a nanoscale conductive tip in combine with a bottom electrode and thus can be rather localized and strong, making it advantages in performing local probing and control on the domain structure. Driven by the application of scanning probe microscope (SFM), in literature, there are quite an amount theoretical works focusing on the stability of nanodomains and domain walls under SPM electric field. In particular, researchers paid attention to dependence of 180° nanodomains stability on SPM electric field (e.g., strength and period) [335,336]. The interaction of ferroelectric 180°-domain wall with inhomogeneous electric field of biased tip has also been analyzed within a phenomenological theory [337]. On the other hand, phase field simulations have been adopted to combine with experiments to explore the mechanism of ferroelectric switching under inhomogeneous electric field. For example, Balke *et al.* [338] suggested method for controlled formation of predefined ferroelectric domains patterns. Later, the spontaneous formation of topological defects at the ferroelastic domain wall during the polarization switching in epitaxial BiFeO_3_ thin films under a biased SFM tip was demonstrated by Vasudevan *et al.* [339]. Recently, Gao *et al.* [340] have presented the dominant role that the pre-existing immobile ferroelastic domain played in the 180° domain switching in PZT thin films. They found that the motion of the 180° ferroelectric domain is hindered by the embedded ferroelastic domain. Overcoming of the hindering effect due to the formation of the transient layer at the interface requires a higher tip field, which prevents the simple, low voltage-switching process.

### 4.4. Ferroelectric Domain Wall and Its Dynamics

Domain walls in UFFs are of fundamental importance and technological relevance, since they are at the core of the polarization switching and make important contributions to most of material responses, thus playing central roles in the practical applications of UFFs such as ultrahigh-density ferroelectric memory, sensors, and actuators, *etc.* In general, the domain walls affect the properties of a ferroelectric thin film in two ways, *i.e.*, the microscopically and macroscopically. In the first case, the existence of domain walls locally modified the properties of thin film, such as local enhancement of piezoelectric responses and increase of conductivity near the domain wall [341]. Even the functionality near the domain wall region has been found to be different from that in the domain region, due to the fact that domain wall can have a different symmetry from those of the domains [342]. In the latter case, we mean that the overall properties of the ferroelectric thin film are influenced by the density and types of domain walls, such as the overall electromechanical responses of thin film can be enhanced by dense ferroelastic domain walls. Another typical example is the bulk photovoltaic effect of ferroelectric thin films caused by the dense domain walls, as has been observed in numerous experimental investigations [343,344,345,346].

To address the issue of domain walls, phenomenological level of description were previously adopted. There were several articles aiming to estimate the thickness of domain wall using a Landau-Ginzburg type of continuum theory [347,348,349,350,351]. Although the models are clear in physics and convenient to solve, the applicability of such approaches are limited. Firstly, some of the parameters needed in the expansion of the free energy functional are difficult to evaluate. Secondly and importantly, domain walls are usually very thin and sharp (typically, the order of lattice constants), which makes the application of the continuum theory questionable; otherwise the domain wall has to be modeled as an interface or boundary, which is usually moveable and thus difficult to be traced. We should always bury in mind these when employing a phenomenological theory in the investigation of domain walls. Thus, it is essential to deal with domain wall issues within the framework of microscopic theory, *i.e.*, first-principles calculations and first-principles derived atomic level methods. Phenomenological models available nowadays are focus on domain walls with reasonably large thickness according to available experimental and theoretical estimates, and UFFs with well-defined free energy [352,353,354,355].

#### 4.4.1. Domain Wall Energy and Configuration

**Figure 17:**
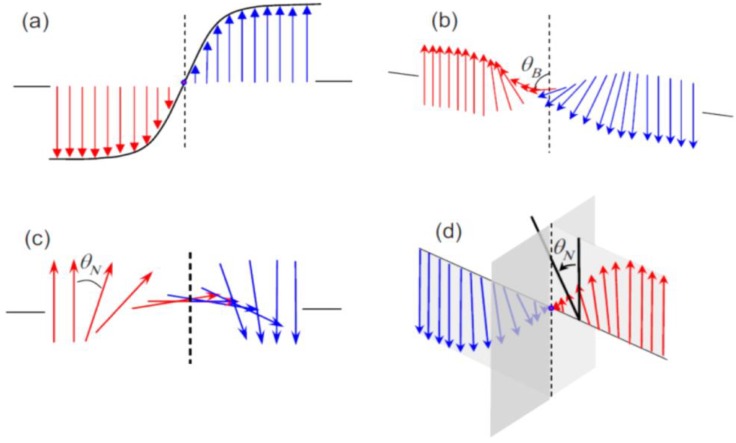
Different types of domain walls: (**a**) Ising type; (**b**) Bloch type; (**c**) Néel type; and (**d**) Mixed Ising-Néel type walls. A mixed Ising-Bloch type would look similar to (**d**) except that the rotation would be out of the plane of the polarization vector. (Reprinted with permission from [355]; Copyright 2009 by the American Physical Society)

**Figure 18:**
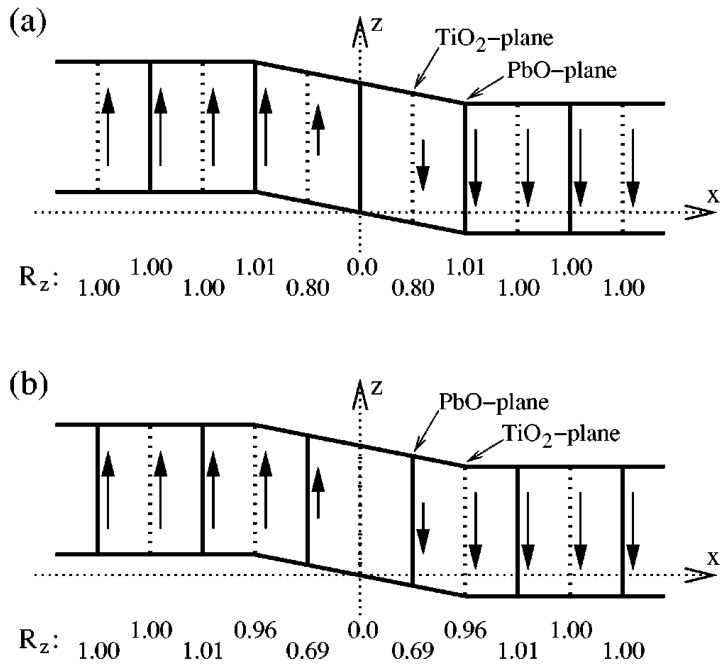
Change of polarization across the (**a**) Pb-centered and (**b**) Ti-centered 180° domain wall. *R_z_* denotes the ferroelectric distortion of each lattice plane in the *z* direction, in units of the distortion associated with the bulk spontaneous polarization. (Reprinted with permission from [173]; Copyright 2002 by the American Physical Society)

The seminal paper by Zhirnov [347] is one of the earliest theoretical investigations of domain wall structures in ferroelectrics based on a phenomenological model. He addressed the difference between ferroelectric domain wall (typically, Ising type, as shown in Figure 17a) and ferromagnets (Bloch type, depicted in Figure 17b), and further predicted the 180° domain wall to be atomically sharp with a width of only 5–20 Å, while the 90° domain wall to be much broader with a thickness of 50–100 Å. Atomistic simulations of domain walls were first performed by Padilla *et al.* [356]. They calculated the energy, free energy and thickness of the 180° walls in tetragonal BaTiO_3_ and confirmed their Ising-like nature along the tetragonal *z* axis employing the effective Hamiltonian method combined with MC simulations at finite temperatures. With first-principles calculations, Pöykkö and Chadi [172,277] studied the 180° domain wall in PbTiO_3_, followed by the investigation of 180° and 90° domain walls in PbTiO_3_ carried out by Meyer and Vanderbilt [173]. These first-principles calculations predicted notable features of domain walls. For (100) 180° domain walls, the A-site centered Ising-type domain wall is most energy favorable. For the 90° domain wall, a much lower domain wall energy being some four times smaller than that of the 180° domain wall was reported. Both the thickness of 180° and 90° walls are of the order of the lattice constant, being very narrow. Figure 18 depicts the configuration of 180° domain walls. Moreover, He and Vanderbilt [278] investigated interaction of oxygen vacancies and 180° domain walls. They predicted that the vacancies do have lower formation energy in the domain wall than in the bulk, thereby confirming the tendency of these defects to migrate to, and pin, the domain walls. More recently, Hlinka and Márton [352] developed a phenomenological model and investigated the 90° domain wall in tetragonal BaTiO_3_-type ferroelectrics. Based on this framework, various domain walls of ferroelectric BaTiO_3_ were further studied. Interestingly, the internal structure of the lowest energy domain wall separating antiparallel rhombohedral ferroelectric domains was shown to be analogous to the so-called Bloch wall known in ferromagnets [353]. Stepkova *et al.* [354] predicted that this antiparallel rhombohedral domain wall can be switched between the Ising-like state (typical for ferroelectrics) and a Bloch-like state by a compressive epitaxial stress. Moreover, Lee *et al.* [355] reported the mixed Bloch-Néel-Ising character of 180° ferroelectric domain walls in prototypical ferroelectrics PbTiO_3_ and LiNbO_3_ (as shown in Figure 17d). Morozovska *et al.* [337] investigated the interaction of ferroelectric 180°-domain wall with a strongly inhomogeneous electric field of biased SPM tip within continuous Ginzburg-Landau-Devonshire theory.

Recently, the structural, electronic, and magnetic properties of the ferroelectric domain walls (*i.e.*, the 71°, 109°, and 180° domain walls) in multiferroic BiFeO_3_ are attracting increasing attention [174,357,358]. Many intriguing characteristics of the domain walls in this multiferroic material, e.g., potential steps and reduction in local band gaps at the 109° and 180° walls, which are correlated with recent measurements electrical conductivity at these boundaries, have been reported [174].

#### 4.4.2. Dynamics of Domain Walls UFFs

**Figure 19:**
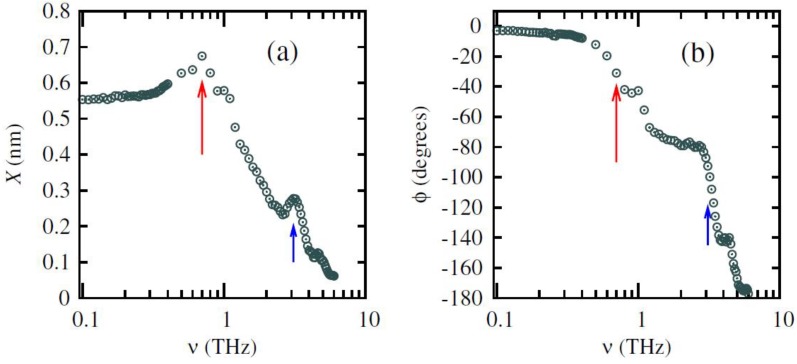
Frequency-dependent response of a nanowall to the electric field in a PZT film. The arrows indicate positions of the peaks. (Reprinted with permission from [280]; Copyright 2011 by the American Physical Society)

Simulations of dynamic behaviors of domain walls in UFFs nowadays are mainly based on MD simulation and effective Hamiltonian combined with MD simulations. Lisenkov *et al.* [279] reported that velocities of the nanodomain walls in ferroelectric BaTiO_3_/SrTiO_3_ superlattices deviate from the common Merz’s law governing the dynamics of larger domains. Furthermore, Zhang and co-workers [280] investigated the nanodynamics of ferroelectric ultrathin films made of PbZr_0.6_Ti_0.4_O_3_ and revealed that the nanodomain walls behaves as an elastic objects and exhibit two types of intrinsic dynamics at the same given frequency (see Figure 19). Based on this, they developed a general theory and proved the size-driven relaxational-resonance dynamics transition in UFFs. Besides, using an effective Hamiltonian method, Ponomareva and Bellaiche [281] investigated the dynamical coupling between polarization and strain pulses of picosecond time-scale in ferroelectric nanolayers, and two different mechanisms were reported. The coupling in homogeneous dipole patterns are found to be governed by the ultrafast soft-mode dynamics, while for inhomogeneous dipole patterns, the coupling occurs through nanobubble’s breathing corresponding to the change of nanobubble size due to the dynamics of the nanobubble wall.

### 4.5. Some Other Effects Related to Domain Structure in UFFs

So far, as presented in the previous sections, domain structures in UFFs under various electrical and mechanical loads have been reviewed, with a main focus on those conventional ferroelectric materials such as BaTiO_3_, PbTiO_3_ and PZT, and on simple electrical and mechanical loads such as homogenous electric field and mechanical strain constraint. As a result, there are some important issues intimately related to domain structure in UFFs being left out. In this Section, we would like to further discuss two important issues, *i.e.*, the flexoelectric effect and the domain structure in multiferroics, which are definitely important but are remaining challenges nowadays for computer simulations.

Flexoelectric effect due to the coupling between polarization and strain gradient are attracting considerable attention quite recently. Such a universal phenomenon which was predicted to be ignorable compared with other effects in solids is of keen interests in UFFs for practical reasons [359,360]: as recent investigations have shown that the flexoelectric coefficients in ferroelectric materials are much larger than originally predicted. Besides, flexoelectricity is a size-dependent phenomenon which is large at the nanoscale because the gradient is inversely proportional to the sample size. Strain gradients in UFFs can be induced by relaxation of the interface strain [361], bending of the underlying substrate, or other external mechanical loads such as pushing an AFM tip on top surface of the film [329]. The flexoelectric effect has been found to act as a built-in field and can be employed in controlling of domain structures in UFFs [329,360,362,363]. However, at present there are only several theoretical investigations of the flexoelectric effects on domain structures [330,364]. For example, using a phase field method, Chen *et al.* [330] explored the mechanical meanings of controlling the stability of 180° nanodomains (*i.e.*, to achieve an effect of mechanical erasing) by mechanical bending strain based on the flexoelectric effect. The difficulty in theoretical calculation resides in lacking of accurate flexoelectric coefficients. Note that the flexoelectric coefficients adopted in most available theoretical investigations nowadays are roughly estimated. Although several attempts based on first-principles calculations to determine the coefficients have been carried out [365,366,367,368], it is far from enough. Many more efforts are urgently needed to figure out to what extent the flexoelectric effect can influence the domain structures in UFFs.

Recently, multiferroics possessing magnetoelectric coupling are quite hot researching objects as they are promising for appearing abundant phenomena and indicate prospective applications. Typical multiferroic materials include *R*Mn_2_O_5_ (*R* = Tb, Dy, Ho, Y, *etc.*), bismuth compounds BiFeO_3_ and BiMnO_3_, and others such as BaNiF_4_, *etc.* Among these multiferroics, perovskite-structured BiFeO_3_, exhibiting ferroelectrictiy, antiferromagnetism, and a weak magnetization resulting from a spin canting at room temperature, is an intriguing example. There have been several theoretical investigations concentrating on the domain structures in BiFeO_3_ thin films. Importantly, using a first-principles-derived effective Hamiltonian method, Prosandeev, Lisenkov and Bellaiche [369] obtained the Kittel law for straight-walled domains in BiFeO_3_ ultrathin films. Based on first-principles calculations, Ren *et al.* [362] investigated the energetic and atomistic characteristics of ferroelectric domains walls of BiFeO_3_ films subject to compressive strain. Despite these efforts, theoretically probing the behaviors of the domains requires a fully understanding of both the ferroelectric and magnetic characteristics. Most simulation methods nowadays are severely limited to the lacking of materials parameters. In particular, in phase field simulation, even more of a problem is the lack of a well-defined free energy describing the magnetic characteristics. Phase field modeling of BiFeO_3_ available are limited to the ferroelectric behaviors.

## 5. Conclusions and Outlook

This paper has reviewed methods and recent developments of theoretical investigations on domain structures in UFFs, aiming to provide readers with the latest knowledge of this field and insights into how factors, e.g., surface, interface, thickness, electrical and mechanical loads, determine the stability and evolution of ferroelectric domain structure and related properties of ultrathin films. It begins with the basic concepts and theories that are relevant to study of domain structures in ultrathin ferroelectric films. Then, it turns to a survey on approaches powerful on domain structure simulation, including first-principles calculation, MD, MC, effective Hamiltonian approach and phase field method. Finally, recent theoretical studies on some important issues of domain structures in ultrathin ferroelectric films are discussed.

To have an outlook of this field, we would like to point out some aspects that are worthy of future attention. 

Firstly, the development of an efficient multiscale investigation scheme is in demand. Among the various theoretical approaches, atomistic level simulations such as MD, MC and effective Hamiltonian, and phase field method are powerful to directly simulate the domain structure with details. Nevertheless, to fully understand the different aspects of domain structure of UFFs, other approaches should be also relied on. In particular, we need first-principles calculations to reveal electronic structure, the accurate energy profile as a function of different degrees of freedom, and detailed physics at the surfaces and interfaces. Such information is necessary for better understanding the behaviors and related properties of domain structures at larger scale. By appropriately choosing thermodynamic variables and construction of free energy, thermodynamic analytic modeling can be an important method of providing a clear description of the thermodynamic behaviors of domain structure. Although in the literature there are some efforts on combining different approaches (mainly first-principles calculations with other approaches), a well-defined and efficient multiscale investigation scheme is lacking. 

Secondly, improving the computational methods to be capable of modeling a wide range of materials, more realistic systems is quite essential in future. Presently, simulations on domain structures in UFFs are mainly performed on conventional perovskite ferroelectrics (e.g., UFFs of perovskite type like BaTiO_3_, PbTiO_3_, and PZT, *etc.*). The investigated boundary conditions and external electrical and mechanical loads are also simple, with a lacking of consideration on complicated situations as well as on the detailed physics of surface and interface. As a consequence, the approaches often only take into account a few degrees of freedom, particularly polarization, strain/stress and electric field. In reality, other degrees of freedom may not be neglected, such as octahedral rotation that is common in perovskite ferroelectric thin films, and magnetic ordering in multiferroic thin films. The incorporation of more degrees of freedom into the theoretical model is definitely important to predict coupling effects and multi-functionalities of domain structure in UFFs.

Moreover, note that currently the true dynamics of domain structure in ferroelectrics is a topic relatively lacking in research, despite its importance in both fundamental research and technological applications. This is largely due to the fact that many approaches, such as MC simulation and phase field method, is not capable of give the true dynamics of domain structures, making them deficient in tackling related problems. On the other hand, MD simulations, as they have the advantage of tracing the intrinsic dynamics of polarization reversal and domain evolution, may be exploited to determine the true kinetic coefficients of the TDGL equation employed in phase field simulations. As the phase field method is capable of dealing large systems and a longer time scale, it would be significant if it can tackle the real dynamics of domain structure. Nevertheless, so far there is no such kind of work being reported. 

Anyway, the field of domain structures in UFFs is now rapidly expanding, with increasing materials and phenomena being under investigation. We are awaiting breakthroughs on theoretical investigations on domain structures in UFFs, particularly on the above mentioned aspects. Although there are many challenges, it is certain that many research efforts will continue to focus on this field. It is quite reasonable to be optimistic that powerful theoretical methods will finally give rise to a systematical understanding of the domain structure in UFFs and provide us instructive information on designing revolutionary devices based on domain structure in UFFs.
